# Predicting Sepsis Mortality in a Population-Based National Database: Machine Learning Approach

**DOI:** 10.2196/29982

**Published:** 2022-04-13

**Authors:** James Yeongjun Park, Tzu-Chun Hsu, Jiun-Ruey Hu, Chun-Yuan Chen, Wan-Ting Hsu, Matthew Lee, Joshua Ho, Chien-Chang Lee

**Affiliations:** 1 Department of Biostatistics Harvard TH Chan School of Public Health Boston, MA United States; 2 Department of Emergency Medicine National Taiwan University Hospital Taipei Taiwan; 3 Department of Internal Medicine Yale School of Medicine New Haven, CT United States; 4 Department of Medicine National Taiwan University Taipei Taiwan; 5 Department of Epidemiology Harvard TH Chan School of Public Health Boston, MA United States; 6 Medical Wizdom, LLC Brookline, MA United States; 7 Center of Intelligent Healthcare National Taiwan University Hospital Taipei Taiwan

**Keywords:** sepsis, mortality, machine learning, SuperLearner

## Abstract

**Background:**

Although machine learning (ML) algorithms have been applied to point-of-care sepsis prognostication, ML has not been used to predict sepsis mortality in an administrative database. Therefore, we examined the performance of common ML algorithms in predicting sepsis mortality in adult patients with sepsis and compared it with that of the conventional context knowledge–based logistic regression approach.

**Objective:**

The aim of this study is to examine the performance of common ML algorithms in predicting sepsis mortality in adult patients with sepsis and compare it with that of the conventional context knowledge–based logistic regression approach.

**Methods:**

We examined inpatient admissions for sepsis in the US National Inpatient Sample using hospitalizations in 2010-2013 as the training data set. We developed four ML models to predict in-hospital mortality: logistic regression with least absolute shrinkage and selection operator regularization, random forest, gradient-boosted decision tree, and deep neural network. To estimate their performance, we compared our models with the Super Learner model. Using hospitalizations in 2014 as the testing data set, we examined the models’ area under the receiver operating characteristic curve (AUC), confusion matrix results, and net reclassification improvement.

**Results:**

Hospitalizations of 923,759 adults were included in the analysis. Compared with the reference logistic regression (AUC: 0.786, 95% CI 0.783-0.788), all ML models showed superior discriminative ability (*P*<.001), including logistic regression with least absolute shrinkage and selection operator regularization (AUC: 0.878, 95% CI 0.876-0.879), random forest (AUC: 0.878, 95% CI 0.877-0.880), xgboost (AUC: 0.888, 95% CI 0.886-0.889), and neural network (AUC: 0.893, 95% CI 0.891-0.895). All 4 ML models showed higher sensitivity, specificity, positive predictive value, and negative predictive value compared with the reference logistic regression model (*P*<.001). We obtained similar results from the Super Learner model (AUC: 0.883, 95% CI 0.881-0.885).

**Conclusions:**

ML approaches can improve sensitivity, specificity, positive predictive value, negative predictive value, discrimination, and calibration in predicting in-hospital mortality in patients hospitalized with sepsis in the United States. These models need further validation and could be applied to develop more accurate models to compare risk-standardized mortality rates across hospitals and geographic regions, paving the way for research and policy initiatives studying disparities in sepsis care.

## Introduction

### Background

Sepsis is a life-threatening condition caused by a dysregulated response of the body to infection. Sepsis is associated with high morbidity and mortality, increased health care expenditures, and long-term consequences [1-4]. It is a leading cause of hospitalization and death, with an estimated 850,000 emergency department visits per year and 59.6 deaths per 100,000 individuals in the United States [2,3]. The annual medical costs associated with sepsis are approximately US $24 billion in the United States [4]. There are clinical and economic incentives to improve and measure the quality of sepsis care in the United States [5]. Given the significant geographic disparities in sepsis outcomes, the development of robust severity adjustment tools is essential for objective sepsis mortality comparisons between hospitals.

Several tools to adjust for sepsis severity have been proposed by consensus conferences [6-8] using traditional statistical methods [9-11]. More recently, machine learning (ML) algorithms have improved the accuracy of sepsis mortality prediction models [12-16]. These tools were largely designed to incorporate the point-of-care risk stratification of patients into the clinical workflow [17-19]. Interhospital comparisons of sepsis care quality and evaluation of risk-adjusted sepsis outcomes have been difficult as the extraction of necessary data from each electronic medical record (EMR) system is time-consuming and not cost-effective [20,21]. Consequently, hospital administrative databases have gradually played a more prominent role and become more widely used by health service researchers because of their easy accessibility and inexpensiveness.

Existing efforts to test and refine sepsis mortality prediction models using hospital administrative data [20-23] have largely used logistic regression models and achieved satisfactory discrimination and calibration. More recent models adjusting for risk factors have made use of national administrative databases to compare risk-adjusted sepsis mortality between hospitals [24]. Although most of them achieved a good area under the receiver operating characteristic curve (AUC) in the range of 0.70-0.80, there is still room to improve their performance. In addition, these studies have focused on select academic centers with limited generalizability to other types of hospitals. Among them, the Severe Sepsis Mortality Prediction Model achieved the best performance with an AUC of 0.838 and was used to generate an integer-based score for risk adjustment in administrative data [21].

### Objectives

ML models have a better ability to automatically select variables, handle large sets of variables, and detect complex multi-way interactions as well as nonlinear relationships [25]. These features enable ML models to improve on conventional regression models in predicting health-related outcomes [26]. In this study, we compare the outcomes of several ML algorithms to predict sepsis mortality using the full range of variables provided in the US Nationwide Inpatient Sample (NIS) database [27,28]. We also determine the accuracy among different derivation and validation models. Our objective is to provide an accurate and reliable tool to compare sepsis-related mortality between hospitals in the United States.

## Methods

### Identification of Cases

Transparent reporting of a multivariable prediction model for individual prognosis or diagnosis was used in this study. The sepsis cohort was identified using the Martin implementation [29,30], which identifies cases with explicit codes from the International Classification of Diseases, Ninth Revision, Clinical Modification for sepsis or systemic fungal infection (038 septicemia, 020.0 septicemic, 790.7 bacteremia, 117.9 disseminated fungal infection, 112.5 disseminated Candida infection, or 112.81 disseminated fungal endocarditis) and a diagnosis of acute organ dysfunction. Seven acute organ or system dysfunctions were evaluated in this study: cardiovascular or shock, respiratory, central nervous system, hematologic, hepatic, renal, and metabolic system dysfunction. To reduce self-prophecy bias, we removed cases with cardiac arrest and ventricular fibrillation, respiratory failure, and respiratory insufficiency.

We split the data into a training set (NIS 2010-NIS 2013) and a testing set (NIS 2014). As the random forest and neural network models could not handle missing values, we removed patients with any missing values from the predictor variables. After removing patients with any missing values, the training data set included 726,918 adult patients, and the validation cohort included 196,841 adult patients.

### Ethical Considerations

Our study involved analysis of de-identified patients from publicly available data. Therefore, no ethics approval was required by the Institutional Review Board (IRB).

### Variables

We used 5-dimensional data as predictors (demographic characteristics, pre-existing comorbidities, hospital characteristics, diagnosis, and procedure performed on the first day of admission). A total of 1331 variables were included in the ML models. We compared our ML models with the reference model using the conventional logistic regression model with predictors reported in a previous study [21]. In the random forest model, we used the Gini Impurity to compute variable importance, where the improvement in the split criterion is the importance attributed to the splitting variable, and identified the top 50 variables based on the variable of importance values [31]. In addition, we calculated the Shapley Additive Explanations (SHAP) values from the xgboost model. SHAP is a popular model-agnostic, local explanation approach designed to explain any given classifier. Lundberg and Lee [32] proposed the SHAP value as a united approach to explaining the output of any ML model. We calculated the SHAP values of each feature for each sample and extracted the top 50 variables based on the mean SHAP values.

### Model Development

We developed four models using ML approaches: (1) logistic regression with least absolute shrinkage and selection operator (LASSO) regularization (LASSO regression), (2) random forest, (3) gradient-boosted decision tree, and (4) deep neural network. In these ML models, we used several methods to minimize potential overfitting in each model: (1) LASSO regularization, (2) out-of-bag estimation, (3) cross-validation, (4) dropout, (5) ridge regularization, and (6) batch normalization.

Finally, we compared our results with the Super Learner model, which is an algorithm that uses cross-validation to estimate the performance of multiple ML models and summarizes the prediction of those models using the ensemble method [33]. In addition, we trained a logistic regression model that used the same features as the ML models. The main analytic script can be found in Multimedia Appendix 1.

### Conventional Logistic Regression (Severe Sepsis Mortality Prediction Model)

Logistic regression uses a function ranging between 0 and 1 to describe the probability that the outcome belongs to one of 2 particular categories. In contrast to linear regression, logistic regression does not require predicted variables to have a linear relationship with the outcome. Logistic regression is well suited for classification problems, such as problems involving describing the risk of developing a disease or the risk of mortality. In this study, we used the previously published Severe Sepsis Mortality Prediction Model as a reference for benchmarking.

### Logistic Regression Model With LASSO Regularization

LASSO regularization is a model that shrinks regression coefficients toward 0, thereby effectively selecting important predictors and improving the interpretability of the model [34]. The coefficients of the LASSO regression are the values that minimize the residual sum of squares plus shrinkage penalty. The regularization was tuned by minimizing *λ* to minimize the mean squared error. We used 10-fold cross-validation to yield the optimal regularization parameter minimizing the sum of least squares plus shrinkage penalty using the R glmnet package (R Foundation for Statistical Computing).

### Random Forest Models

Random forest is an ensemble of decision trees from bootstrapped training samples. Random forests modify the bagged tree procedure by only allowing a random number of the predictor variables to be considered at each split of each tree [26,35,36]. For this study, the Gini Impurity was used to determine the optimal variable and location of the split at each node in the tree. To optimize the AUC of the resulting tree, a cost complexity parameter, which penalizes larger trees, was used to control the size of the final tree. To improve the accuracy and stability of the decision tree model, a procedure called bagging was used to fit a bagged tree model [37]. This involved taking random bootstrap samples of patient data with replacement and fitting an unpruned tree model to each sample. The number of bagged trees in the final model was determined in the training data set using 10-fold cross-validation to maximize the training set AUC. We considered from 100 to 2000 trees and performed a pairwise statistical test to choose the best number of trees (Table 1). This results in trees that are less correlated with each other compared with bagged trees, thus potentially increasing accuracy. The optimal number of trees and predictor variables to be considered at each split was determined using 10-fold cross-validation, and the combination with the highest training set AUC was denoted as the final model. Table 2 shows the association between the number of variables allowed to be considered at each split in the random forest model with discrimination. The final random forest model was fitted with 400 trees with 50 variables at each split. We used the ranger package in R to construct the random forest models.

**Table 1:** Sensitivity analysis of tree numbers in the random forest algorithm.

Number of trees allowed	AUC^a^ (95% CI)	Pairwise significant comparison of AUC	*P* value
100	0.876 (0.874-0.878)	100 trees versus 200 trees	<.001^b^
200	0.877 (0.876-0.879)	200 trees versus 300 trees	<.001^b^
300	0.878 (0.877-0.880)	300 trees versus 400 trees	<.001^b^
400	0.878 (0.877-0.880)	400 trees versus 500 trees	.30

^a^AUC: area under the curve.

^b^Values are significant at *P*<.001.

**Table 2:** Association between the number of variables allowed to be considered at each split in the random forest model and model discrimination.

Number of variables allowed	AUC^a^ (95% CI)	Number of variables	Pairwise significant comparison of AUC (*P* value)
3	0.852 (0.850-0.854)	3 variables versus 5 variables	<.001^b^
5	0.860 (0.858-0.862)	5 variables versus 9 variables	<.001^b^
9	0.868 (0.866-0.869)	9 variables versus 15 variables	<.001^b^
15	0.874 (0.872-0.875)	15 variables versus 20 variables	<.001^b^
20	0.875 (0.874-0.877)	20 variables versus 25 variables	<.001^b^
25	0.877 (0.875-0.879)	25 variables versus 40 variables	<.001^b^
40	0.878 (0.876-0.880)	40 variables versus 50 variables	.02^c^
50	0.878 (0.877-0.880)	50 variables versus 70 variables	.53
70	0.878 (0.877-0.880)	N/A^d^	N/A

^a^AUC: area under the curve.

^b^Values are significant at *P*<.001.

^c^Values are significant at *P*<.05.

^d^N/A: not applicable.

### Xgboost

Gradient-boosted decision trees are also an ensemble method that constructs new tree models predicting the errors and residuals of previous models [38]. When adding the new models, this model uses a gradient descent algorithm to minimize the loss function. The final tree-based model fit was a gradient-boosted machine. This algorithm fits one tree at a time, first to all the outcomes in the training data and then to the residuals of the previous models, thus creating a combination of trees that increasingly weigh the *difficult to predict* events to a greater degree. The optimal number of splits for each individual tree, the total number of trees, and the learning rate were determined using 10-fold cross-validation in a similar method to that of the random forest model. In our final model, we had 10 splits for each tree in a total of 400 trees with a learning rate of 0.15. We stopped training if the validation AUC did not improve in 3 epochs. We used the xgboost package in R to construct the gradient-boosted decision tree models.

### Deep Neural Networks Keras

**Figure 1:**
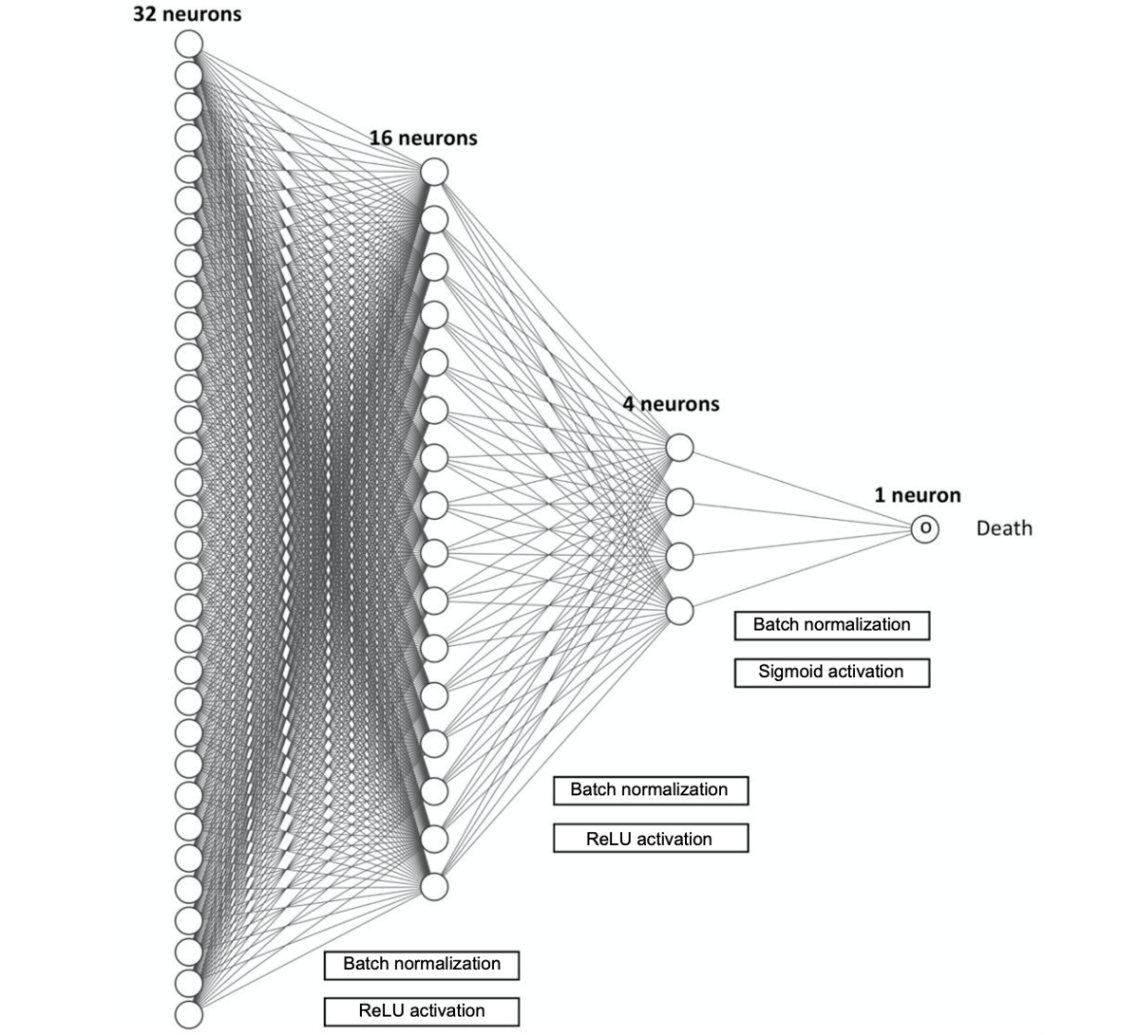
The architecture of the 4-layered neural network to predict sepsis mortality. ReLu: Rectified Linear Unit.

Deep neural network models are composed of multiple processing layers. Neural networks are nonlinear models that involve creating a set of linear combinations of the original predictor variables and then using them as inputs into a hidden layer (or layers) of units, which then creates new combinations of these inputs to finally output the probability of the event of interest after a suitable transformation [39]. A feedforward multilayer perceptron neural network was used for this study. A penalty term, known as weight decay, and the number of hidden units in the model were determined using 10-fold cross-validation to maximize the training set AUC. We used a 4-layer feedforward model with an adaptive moment estimation optimizer, the binary cross-entropy loss function, and tuned hyperparameters using the R keras package. In the neural network model, continuous predictors are normalized using the mean and SDs. Binary variables encoding 0 and 1 are rescaled to encode −1 and 1. Finally, categorical variables use rescaled using effect encodings. The detailed architecture of the deep neural network in this study is shown in Figure 1.

### Super Learner

Finally, we compared our results with the Super Learner algorithm, which uses cross-validation to estimate the performance of multiple ML models [33]. The Super Learner takes all weighted combinations from a set of candidate algorithms. After a set of algorithms is chosen, the meta-learning algorithm performs cross-validation to estimate the maximum likelihood of each selected algorithm on the data and selects the convex combination with the smallest squared prediction error on the test data set. In our case, we chose logistic regression as our meta-model. Overall, the generalization procedure learns the *n*-fold stratified predictions to maximize the likelihood function rather than minimize the mean squared error and to represent the meta-model in generating the best prediction. This approach has been proven to be as accurate as the best possible prediction algorithm. We used the 2 algorithms (random forest and xgboost) that we considered in this manuscript as candidate algorithms and compared the results with the models discussed in this paper. Our Super Learner scripts can be found in Multimedia Appendices 2-4.

### Model Performance

In the test set (NIS 2014), we computed the prediction performance of each model that was derived above. First, we calculated the area under the receiver operating characteristic curve (AUROC) and confusion matrix results. The Delong test was used to compare the receiver operating characteristic curves between models. Second, the confusion matrix results were calculated. Third, given the imbalanced nature of our data set, we also calculated the area under the precision-recall curve (AUC-PR), recall, and precision of different ML models in predicting sepsis mortality. Fourth, calibration curves were constructed by plotting predicted probability versus actual probability from the ML models. The Brier scores of all the models considered were also calculated. The Brier score is a quadratic scoring rule where the squared differences between the actual binary outcomes and predicted probabilities are calculated. Therefore, lower values indicate better calibration. All analyses were performed using R (version 3.6.1).

### Web Application

To increase the reproducibility and usability of this research on sepsis care and mortality, we generated a web-based application [40] for peer investigators to generate predictions of 30-day mortality for patients with sepsis and provide an introductory video (Multimedia Appendix 2). The web application is based on our Super Learner model and built using the Shiny package in R (version 4.0.5). The submission interface offers an example Microsoft Excel file with placeholder columns. Details of how to generate the variables are described in Multimedia Appendix 5.

## Results

### Baseline Characteristics

**Figure 2:**
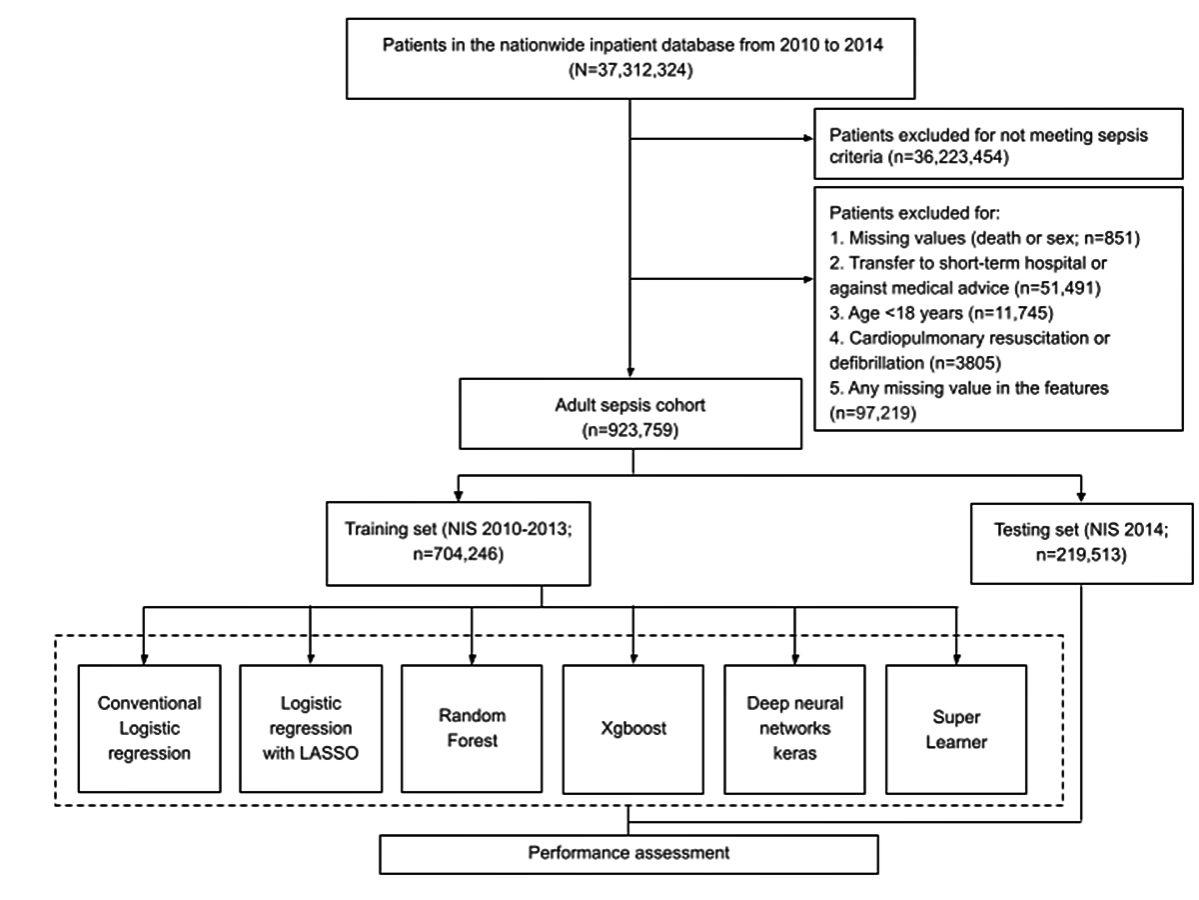
Flowchart depicting the construction of the study cohort from the Nationwide Inpatient Sample (NIS) database. LASSO: least absolute shrinkage and selection operator.

Figure 2 shows the flowchart of the cohort used in this study, and Table 3 provides descriptive statistics of survivors and nonsurvivors of sepsis from the cohort used. Table 4 shows the characteristics of patients with sepsis stratified by training and validation cohort.

**Table 3:** Characteristics of patients with sepsis in the Nationwide Inpatient Sample stratified by in-hospital survival status (N=923,759).

Characteristics		Survivors of sepsis (n=726,918)	Nonsurvivors of sepsis (n=196,841)	Total
Age (years), mean (SE)		67.15 (16.44)	70.85 (14.88)	67.94 (16.19)
Women, n (%)		358,756 (49.4)	96,708 (49.1)	455,464 (49.3)
**Race, n (%)**				
	White	511,579 (70.4)	137,807 (70)	649,386 (70.3)
	Black	112,801 (15.5)	30,207 (15.3)	143,008 (15.5)
	Hispanic	61,174 (8.4)	16,386 (8.3)	77,560 (8.4)
	Others	41,364 (5.7)	12,441 (6.3)	53,805 (5.8)
**Insurance, n (%)**				
	Medicare	221,228 (30.4)	60,933 (31)	282,161 (30.5)
	Medicaid	185,758 (25.6)	48,838 (24.8)	234,596 (25.4)
	Commercial	172,650 (23.8)	45,437 (23.1)	218,087 (23.6)
	Other	147,282 (20.3)	41,633 (21.2)	188,915 (20.5)
**Measures of acute illness severity, n (%)**				
	Early mechanical ventilation	118,939 (16.4)	76,773 (39)	195,712 (21.2)
	Late mechanical ventilation	36,649 (5)	35,531 (18.1)	72,180 (7.8)
	Shock	305,375 (42)	132,582 (67.4)	437,957 (47.4)
	Hemodialysis	58,962 (8.1)	28,691 (14.6)	87,653 (9.5)
	ICU^a^ care (at least one day)	67,810 (9.3)	58,756 (29.8)	126,566 (13.7)
**Underlying comorbidity, n (%)**				
	Anemia	265,364 (36.5)	55,632 (28.3)	320,996 (34.7)
	Depression	81,827 (11.3)	14,612 (7.4)	96,439 (10.4)
	Diabetes	256,947 (35.3)	57,294 (29.1)	314,241 (34)
	Drug and substance abuse	25,311 (3.5)	4188 (2.1)	29,499 (3.2)
	Chronic lung disease	188,546 (25.9)	50,749 (25.8)	239,295 (25.9)
	Congestive heart failure	173,776 (23.9)	56,036 (28.5)	229,812 (24.9)
	Hypertension	424,834 (58.4)	102,862 (52.3)	527,696 (57.1)
	Hypothyroid disease	100,256 (13.8)	23,856 (12.1)	124,112 (13.4)
	Liver disease	42,065 (5.8)	17,995 (9.1)	60,060 (6.5)
	Renal failure, chronic	210,371 (28.9)	57,171 (29)	267,542 (29)
	Lymphoma	13,691 (1.9)	5469 (2.8)	19,160 (2.1)
	Metastatic carcinomas	30,789 (4.2)	17,109 (8.7)	47,898 (5.2)
	Neurological conditions	117,134 (16.1)	27,791 (14.1)	144,925 (15.7)
	Obesity	100,716 (13.9)	18,173 (9.2)	118,889 (12.9)
	Malignant solid tumors	27,426 (3.8)	10,057 (5.1)	37,483 (4.1)
	Rheumatoid arthritis or collagen vascular diseases	27,294 (3.8)	6324 (3.2)	33,618 (3.6)
	Paraplegia	53,755 (7.4)	10,955 (5.6)	64,710 (7)
	Perivascular conditions	68,641 (9.4)	22,853 (11.6)	91,494 (9.9)
	Psychiatric diseases	44,282 (6.1)	6902 (3.5)	51,184 (5.5)
	Pulmonary-circulatory	43,697 (6)	15,327 (7.8)	59,024 (6.4)
	Weight loss	146,865 (20.2)	47,320 (24)	194,185 (21)
**System dysfunction, n (%)**				
	Renal dysfunction	433,920 (59.7)	129,768 (65.9)	563,688 (61)
	Cardiovascular dysfunction or shock	281,647 (38.7)	132,079 (67.1)	413,726 (44.8)
	Acute respiratory failure	161,921 (22.3)	116,406 (59.1)	278,327 (30.1)
	CNS^b^ dysfunction	162,716 (22.4)	51,146 (26)	213,862 (23.2)
	Hepatic dysfunction	18,579 (2.6)	20,561 (10.4)	39,140 (4.2)
**Lifestyle factors, n (%)**				
	Smoking	75,404 (10.4)	15,033 (7.6)	90,437 (9.8)
	Alcoholism	32,879 (4.5)	10,674 (5.4)	43,553 (4.7)

^a^ICU: intensive care unit.

^b^CNS: central nervous system.

**Table 4:** Characteristics of patients with sepsis in the Nationwide Inpatient Sample stratified by training and validation cohort (N=923,759).

Characteristic			Training (2010-2013)					Testing (2014)	
			Survivors of sepsis (n=548,930)		Nonsurvivors of sepsis (n=155,316)		Survivors of sepsis (n=177,988)		Nonsurvivors of sepsis (n=41,525)
Age (years), mean (SE)			67.25 (16.46)		70.96 (14.93)		66.84 (16.37)		70.44 (14.68)
Women, n (%)			271,311 (49.4)		76,496 (49.3)		87,445 (49.1)		20,212 (48.7)
**Race, n (%)**									
	White	385,330 (70.2)		108,405 (69.8)		126,249 (70.9)			29,402 (70.8)
	Black	86,727 (15.8)		24,295 (15.6)		26,074 (14.6)			5912 (14.2)
	Hispanic	45,887 (8.4)		12,954 (8.3)		15,287 (8.6)			3432 (8.3)
	Others	30,986 (5.6)		9662 (6.2)		10,378 (5.8)			2779 (6.7)
**Insurance, n (%)**									
	Medicare	166,023 (30.2)		47,814 (30.8)		55,205 (31)			13,119 (31.6)
	Medicaid	136,607 (24.9)		37,627 (24.2)		49,151 (27.6)			11,211 (27)
	Commercial	132,428 (24.1)		36,387 (23.4)		40,222 (22.6)			9050 (21.8)
	Other	113,872 (20.7)		33,488 (21.6)		33,410 (18.8)			8145 (19.6)
**Measures of acute illness severity, n (%)**									
	Early mechanical ventilation	92,718 (16.9)		60,822 (39.2)		26,221 (14.7)			15,951 (38.4)
	Late mechanical ventilation	28,892 (5.3)		28,532 (18.4)		7757 (4.4)			6999 (16.9)
	Shock	232,963 (42.4)		103,544 (66.7)		72,412 (40.7)			29,038 (69.9)
	Hemodialysis	46,180 (8.4)		22,818 (14.7)		12,782 (7.2)			5873 (14.1)
	ICU^a^ care (at least one day)	53,146 (9.7)		46,914 (30.2)		14,664 (8.2)			11,842 (28.5)
**Underlying comorbidity, n (%)**									
	Anemia	201,132 (36.6)		43,380 (27.9)		64,232 (36.1)			12,252 (29.5)
	Depression	59,998 (10.9)		11,239 (7.2)		21,829 (12.3)			3373 (8.1)
	Diabetes	191,296 (34.8)		44,598 (28.7)		65,651 (36.9)			12,696 (30.6)
	Drug and substance abuse	17,689 (3.2)		3113 (2)		7622 (4.3)			1075 (2.6)
	Chronic lung disease	140,276 (25.6)		39,550 (25.5)		48,270 (27.1)			11,199 (27)
	Congestive heart failure	130,913 (23.8)		43,716 (28.1)		42,863 (24.1)			12,320 (29.7)
	Hypertension	316,301 (57.6)		79,939 (51.5)		108,533 (61)			22,923 (55.2)
	Hypothyroid disease	73,904 (13.5)		18,348 (11.8)		26,352 (14.8)			5508 (13.3)
	Liver disease	30,753 (5.6)		13,796 (8.9)		11,312 (6.4)			4199 (10.1)
	Renal failure, chronic	158,078 (28.8)		44,704 (28.8)		52,293 (29.4)			12,467 (30)
	Lymphoma	10,371 (1.9)		4281 (2.8)		3320 (1.9)			1188 (2.9)
	Metastatic carcinomas	23,087 (4.2)		13,352 (8.6)		7702 (4.3)			3757 (9)
	Neurological conditions	87,994 (16)		21,699 (14)		29,140 (16.4)			6092 (14.7)
	Obesity	71,693 (13.1)		13,392 (8.6)		29,023 (16.3)			4781 (11.5)
	Malignant solid tumors	20,417 (3.7)		7814 (5)		7009 (3.9)			2243 (5.4)
	Rheumatoid arthritis or collagen vascular diseases	20,368 (3.7)		4898 (3.2)		6926 (3.9)			1426 (3.4)
	Paraplegia	40,811 (7.4)		8488 (5.5)		12,944 (7.3)			2467 (5.9)
	Perivascular conditions	50,853 (9.3)		17,734 (11.4)		17,788 (10)			5119 (12.3)
	Psychiatric diseases	32,698 (6)		5320 (3.4)		11,584 (6.5)			1582 (3.8)
	Pulmonary-circulatory	32,214 (5.9)		11,625 (7.5)		11,483 (6.5)			3702 (8.9)
	Weight loss	113,028 (20.6)		37,182 (23.9)		33,837 (19)			10,138 (24.4)
**System dysfunction, n (%)**									
	Renal dysfunction	324,840 (59.2)		101,420 (65.3)		109,080 (61.3)			28,348 (68.3)
	Cardiovascular dysfunction or shock	215,545 (39.3)		103,064 (66.4)		66,102 (37.1)			29,015 (69.9)
	Acute respiratory failure	125,706 (22.9)		92,008 (59.2)		36,215 (20.3)			24,398 (58.8)
	CNS^b^ dysfunction	118,837 (21.6)		38,642 (24.9)		43,879 (24.7)			12,504 (30.1)
	Hepatic dysfunction	14,091 (2.6)		15,752 (10.1)		4488 (2.5)			4809 (11.6)
**Lifestyle factors, n (%)**									
	Smoking	54,038 (9.8)		11,205 (7.2)		21,366 (12)			3828 (9.2)
	Alcoholism	24,025 (4.4)		8083 (5.2)		8854 (5)			2591 (6.2)

^a^ICU: intensive care unit.

^b^CNS: central nervous system.

### Performance Comparison

**Figure 3:**
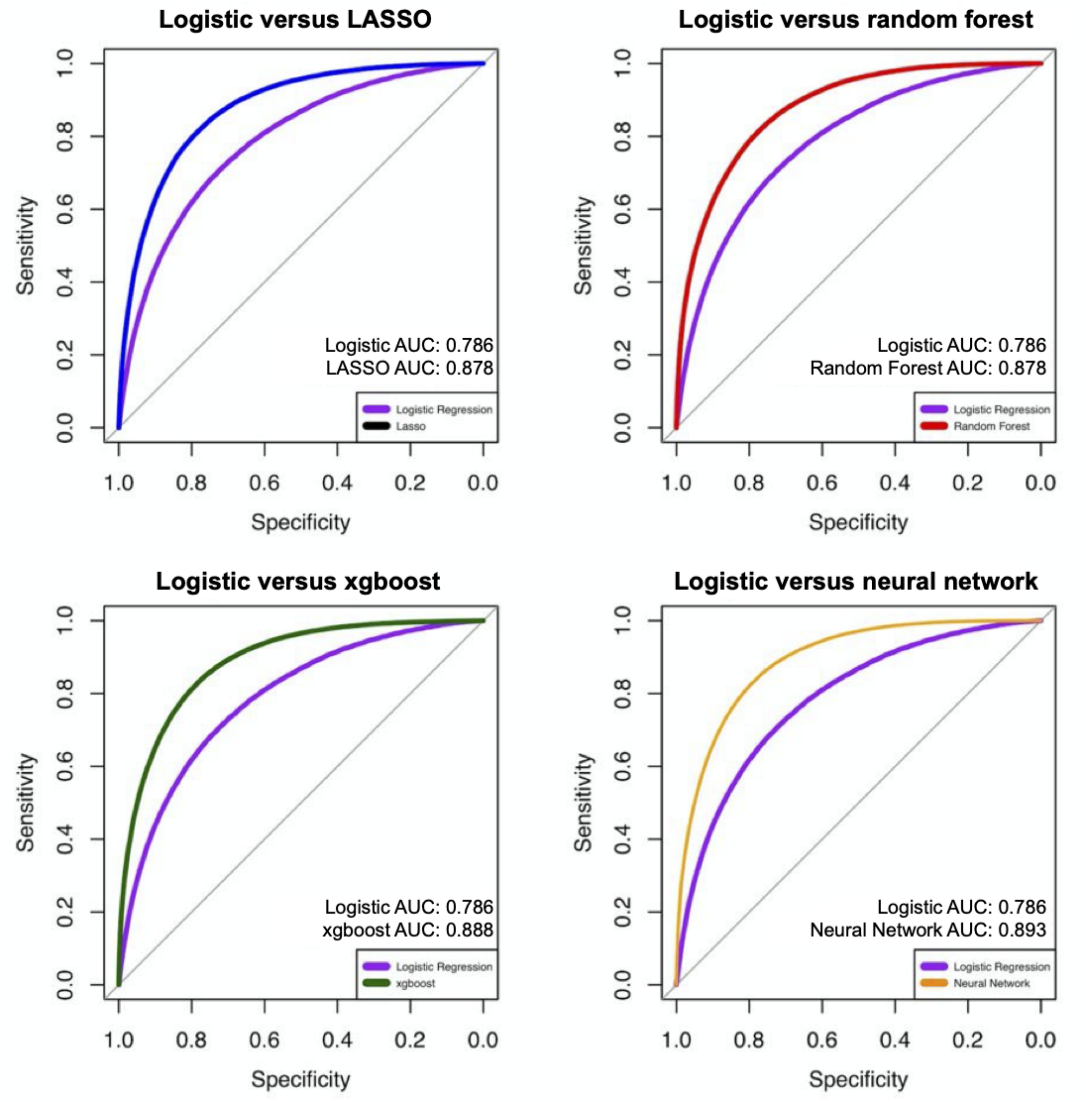
Receiver operating characteristic curves of different machine learning models in predicting sepsis mortality. AUC: area under the curve; LASSO: least absolute shrinkage and selection operator.

**Figure 4:**
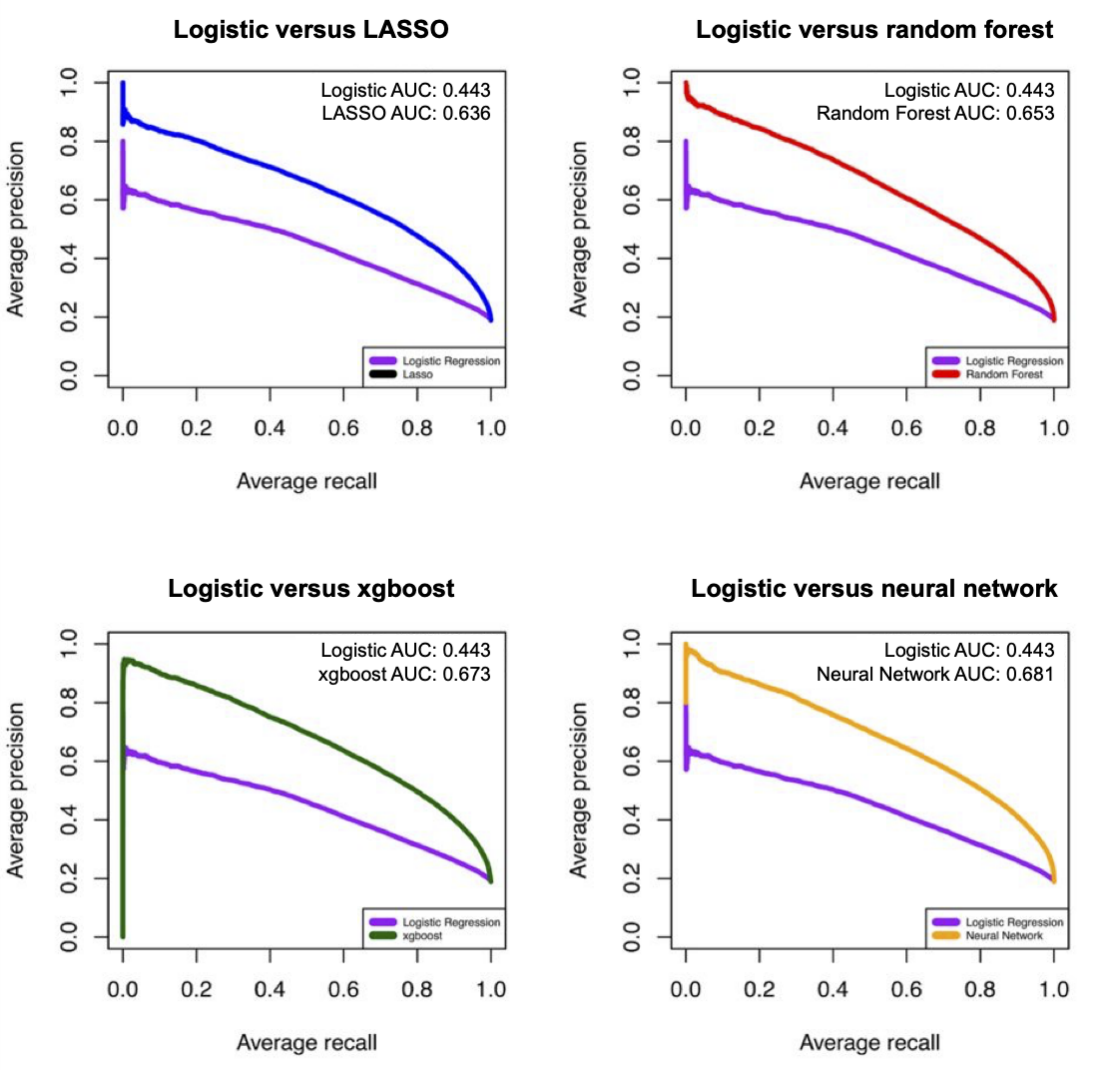
Precision-recall curves of different machine learning models in predicting sepsis mortality. AUC: area under the curve; LASSO: least absolute shrinkage and selection operator.

Compared with the reference logistic regression model (0.786, 95% CI 0.783-0.788), all 4 ML methods showed superior discriminative ability (*P*<.001; Table 5). Of all 4 ML methods, the deep neural network showed the highest (*P*<.001) discriminative ability (0.893, 95% CI 0.891-0.895) followed by the gradient-boosting model (0.888, 95% CI 0.886-0.889). The AUC of the deep neural network (0.893, 95% CI 0.891-0.895) was higher than that of the Super Learner model (0.883, 95% CI 0.881-0.885). Both LASSO (0.878, 95% CI 0.876-0.879) and random forest (0.878, 95% CI 0.877-0.880) had an AUC that was slightly lower among the ML models but was nevertheless superior (*P*<.001) to the reference logistic model (Figures 3 and 4).

Of the ML models, the deep neural network also demonstrated higher specificity (0.794, 95% CI 0.793-0.796) and positive predictive value (0.484, 95% CI 0.480-0.488) while resulting in lower sensitivity (0.826, 95% CI 0.823-0.830) and negative predictive value (0.951, 95% CI 0.950-0.953) compared with the xgboost model, but these differences were statistically insignificant. The Super Learner showed similar results to our xgboost model, with statistically lower specificity (0.769, 95% CI 0.768-0.771) and positive predictive value (0.458, 95% CI 0.455-0.460) compared with the neural network model. However, the neural network model showed only marginally lower sensitivity (0.826, 95% CI 0.823-0.830) and negative predictive value (0.951, 95% CI 0.950-0.953) compared with the Super Learner.

The AUC-PR, recall, and precision of different ML models in predicting sepsis mortality are shown in Figure 4 and Table 6. The ML models showed superior AUC-PR measures (0.636-0.681) compared with the reference logistic regression model (0.442). In addition, being paralleled with our finding from the AUROC, the deep neural network model showed the highest AUC-PR (0.681) followed by the xgboost model (0.673).

**Figure 5:**
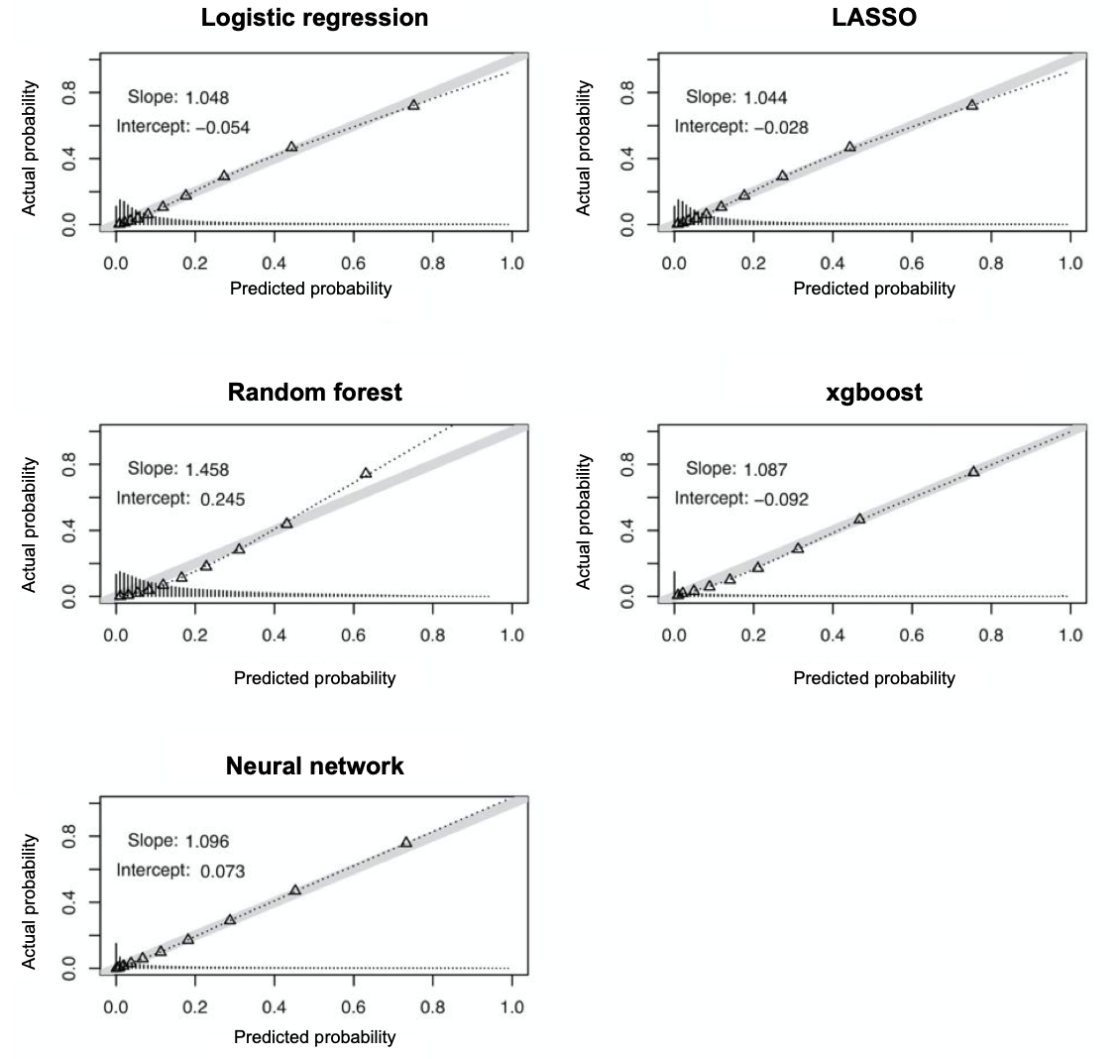
Calibration plots of observed versus predicted hospital mortality and associated mortality ratios by risk deciles in the development and validation cohorts. LASSO: least absolute shrinkage and selection operator.

Most of the models showed great calibration from a visual representation, which shows calibration plots characterized by visual inspection and reporting of the intercept and slope (Figure 5). The intercept’s deviation from 0 indicates the extent to which predictions are underpredicting or overpredicting the probability of the event of interest—sepsis mortality. All of our models showed small departures (intercept <0.1) except for the random forest model, which overpredicted sepsis mortality (0.245). The random forest and neural network models slightly overpredicted sepsis mortality, whereas the reference logistic regression, LASSO, and xgboost models slightly underpredicted sepsis mortality. Compared with the reference logistic regression model, which had a slope of 1.048, LASSO (1.044), xgboost (1.087), and the neural network model (1.096) had similar slopes that were all close to 1. However, the random forest model showed the largest deviation from perfect calibration (1.458).

In addition, Table 7 shows the Brier scores of all the models. The deep neural network model exhibited the lowest Brier score of 0.954 followed by xgboost (0.102), which is in alignment with their high discriminatory ability. The ML models exhibited a good range of Brier scores (0.095-0.108), all of which were higher than those of the reference logistic regression model (0.129).

**Table 5:** Measures of model discrimination and accuracy in the validation data set (Nationwide Inpatient Sample 2014), including area under the curve (AUC), sensitivity, specificity, positive predictive value (PPV), and negative predictive value (NPV).

Model	AUC (95% CI)	Sensitivity (95% CI)	Specificity (95% CI)	PPV (95% CI)	NPV (95% CI)
Reference logistic regression (Severe Sepsis Prediction score)	0.786 (0.783-0.788)	0.708 (0.704-0.713)	0.722 (0.720-0.774)	0.373 (0.370-0.376)	0.914 (0.912-0.915)
LASSO^a^	0.878 (0.876-0.879)	0.812 (0.808-0.816)	0.784 (0.782-0.786)	0.468 (0.464-0.471)	0.947 (0.946-0.948)
Random forest	0.878 (0.877-0.880)	0.818 (0.814-0.821)	0.771 (0.769-0.773)	0.454 (0.451-0.458)	0.948 (0.947-0.949)
Xgboost	0.888 (0.886-0.889)	0.829 (0.826-0.833)	0.781 (0.781-0.785)	0.472 (0.468-0.475)	0.952 (0.950-0.953)
Deep neural network	0.893 (0.891-0.895)	0.826 (0.823-0.830)	0.794 (0.793-0.796)	0.484 (0.480-0.488)	0.951 (0.950-0.953)
Super Learner	0.883 (0.881-0.885)	0.833 (0.829-0.837)	0.769 (0.768-0.771)	0.458 (0.455-0.460)	0.952 (0.951-0.953)

^a^LASSO: least absolute shrinkage and selection operator.

**Table 6:** The area under the precision-recall curve (AUC-PR), recall, and precision of different machine learning models in predicting sepsis mortality.

	AUC-PR, mean (SD)	Recall (95% CI)	Precision (95% CI)
Reference logistic regression	0.443 (0.003)	0.587 (0.583-0.591)	0.403 (0.401-0.405)
LASSO^a^	0.636 (0.001)	0.806 (0.805-0.807)	0.410 (0.410-0.411)
Random forest	0.653 (0.002)	0.806 (0.805-0.807)	0.415 (0.414-0.416)
Xgboost	0.673 (0.002)	0.814 (0.813-0.816)	0.420 (0.420-0.421)
Neural networks	0.681 (0.002)	0.815 (0.814-0.816)	0.427 (0.426-0.428)

^a^LASSO: least absolute shrinkage and selection operator.

**Table 7:** Calibration measures of different machine learning models in predicting sepsis mortality.

	Brier score	Slope	Intercept
Reference logistic regression	0.129	1.048	−0.054
LASSO^a^	0.108	1.044	−0.028
Random forest	0.103	1.458	0.245
Xgboost	0.102	1.087	−0.092
Neural networks	0.0954	1.096	0.073

^a^LASSO: least absolute shrinkage and selection operator.

### Variable Importance of the Random Forest by Gini Impurity and Xgboost Model by SHAP

**Figure 6:**
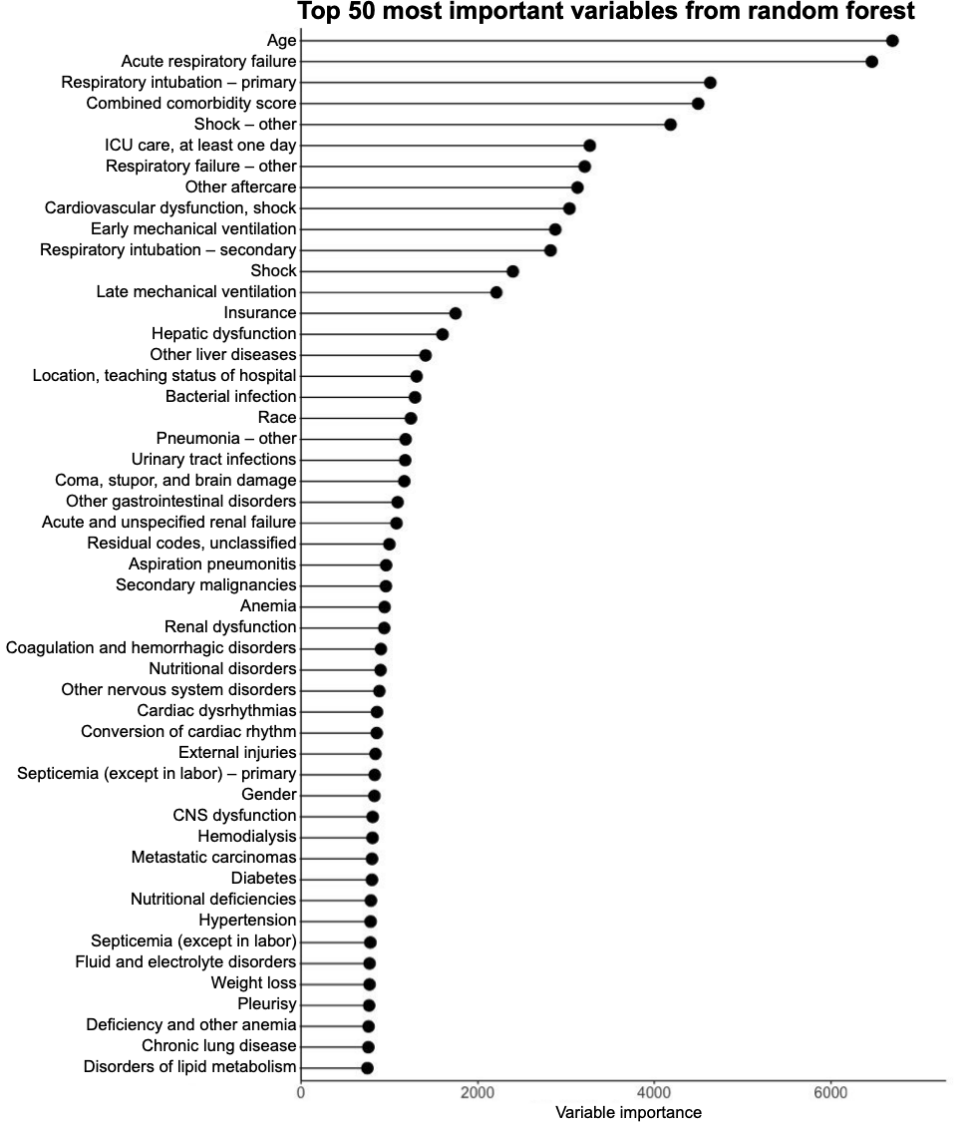
Variables of importance from random forest ranked by impurity-based variable importance. CNS: central nervous system; ICU: intensive care unit.

**Figure 7:**
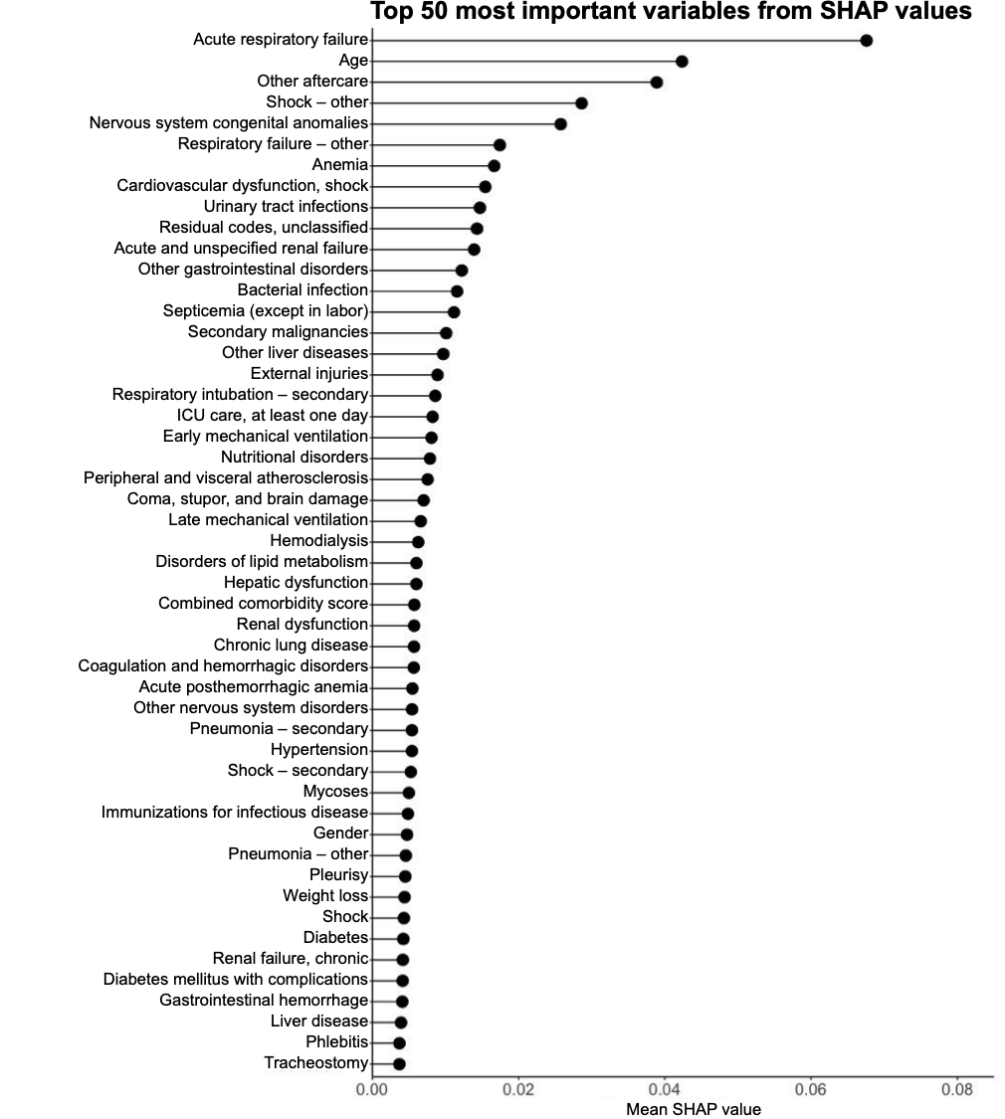
Variables of importance from xgboost ranked by mean Shapley Additive Explanations values (SHAP). ICU: intensive care unit.

**Figure 8:**
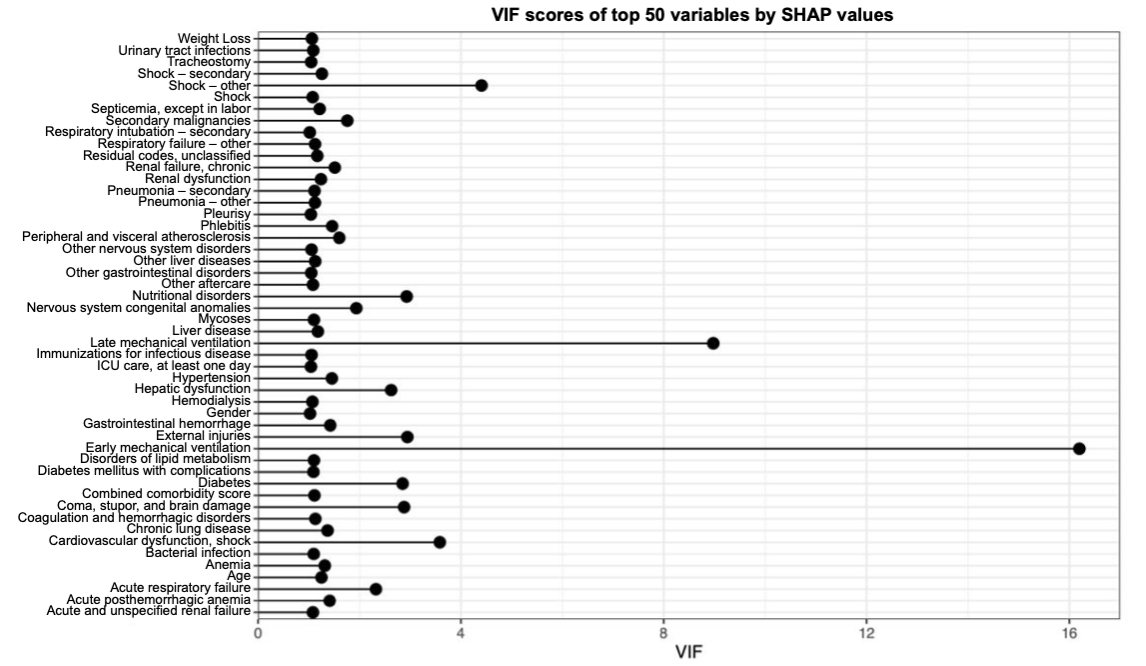
Variance inflation factor scores of top 50 variables by Shapley Additive Explanations (SHAP) values. ICU: intensive care unit; VIF: variance inflation factor.

The top 50 variables according to the variable importance of the random forest algorithm by the Gini Impurity are shown in Figure 6. The top 50 features with the highest mean SHAP values of the xgboost algorithm are shown in Figure 7. SHAP is a popular technique used to explain model predictions. SHAP is model-agnostic, with the ability to explain any given classifier. Lundberg and Lee [32] proposed SHAP as a united approach to explaining the output of any ML model. Acute respiratory failure and age were the 2 most important features from the random forest model as well as the xgboost model, and acute respiratory failure was not a feature in the reference logistic regression model. In addition, we found many diagnosis (primary, secondary, and other) and procedure (primary and secondary) variables to be important predictors for sepsis mortality, which were not included in the reference logistic regression model. To assess collinearity, variance inflation factors of the final feature panel from SHAP were calculated from a total cohort combining both the training and validation cohorts. All 50 features showed variance inflation factor scores <5 except for early mechanical ventilation and late mechanical ventilation (Figure 8).

### Logistic Regression Model Using the Same Features as the ML Models

Moreover, Table 8 shows the performance of a logistic regression model that used the same features as the ML models. Overall, this logistic regression model with all features showed a very comparable calibration performance assessed by the Brier score (0.102) compared with those of the ML models (95% CI 0.0954-0.108). This model resulted in a slightly lower AUC-PR (0.634) compared with the ML models (95% CI 0.636-0.681) and a statistically lower (*P*<.001) AUROC of 0.857 (95% CI 0.855-0.859) compared with the ML models (95% CI 0.876-0.895).

**Table 8:** Performance comparison of the machine learning models with the logistic regression model with the same features.

	Brier score	AUC-PR^a^, mean (SD)	AUC^b^ (95% CI)	AUC *P* value
Logistic regression model—all features	0.102	0.634 (0.003)	0.857 (0.855-0.859)	N/A^c^
LASSO^d^	0.108	0.636 (0.001)	0.878 (0.876-0.879)	<.001^e^
Random forest	0.103	0.653 (0.002)	0.878 (0.877-0.880)	<.001^e^
Xgboost	0.102	0.673 (0.002)	0.888 (0.886-0.889)	<.001^e^
Neural networks	0.0954	0.681 (0.002)	0.893 (0.891-0.895)	<.001^e^

^a^AUPRC: area under the precision-recall curve.

^b^AUC: area under the curve.

^c^N/A: not applicable.

^d^LASSO: least absolute shrinkage and selection operator.

^e^Values are significant at *P*<.001.

Later, we trained a reference logistic regression model and a random forest model using this experiment and compared their performance to that of our original study design. I Instead of splitting our cohort by year, we split the training and testing cohorts randomly to see if our findings hold true. Of the patients from the entire data set, 75% (692,819) were assigned to the training set, and the remaining 25% (230,940) of the samples were assigned to the testing set. Using this approach, we obtained AUROC of 0.765 (95% CI 0.763-0.768) from the reference logistic regression model, whereas we observed superior performance from a random forest model with AUROC of 0.855 (95% CI 0.853-0.857). This finding is consistently with the results with our original approach of splitting our cohort by year where ML models showed superior discrimination performance compared with the reference logistic model.

Finally, we used the Gini Impurity to calculate the variable of importance in our random forest model. In Table 9, we present the results of an analysis of the top 50 most important predictive features when a different train–test split method is used. When our cohort was split randomly, Table 9 shows the top 50 most important features from a random forest model. The third column shows whether these features were also in the top 50 in the previous random forest model using the train–test split-by-year approach (Figure 4). Of the 50 most important features, 44 (88%) were also top features identified by the previous random forest model using the train–test split-by-year approach (Figure 4). Although 6 features have changed, we note that 5 (83%) are low-ranking features with higher variability. As a result, despite having used 2 different train–test split approaches, the features identified and ranked in the top 50 most important features by both models had relatively consistent ranks (ie, the most important features were age and acute respiratory failure).

**Table 9:** Variables of importance from the random forest (random train–test split cohort).

Variable name	Importance rank	Top 50 from previous cohort
Acute respiratory failure	1	Yes
Age	2	Yes
Respiratory intubation and mechanical ventilation (primary procedure)	3	Yes
Combined comorbidity score	4	Yes
Shock (other diagnosis)	5	Yes
ICU^a^ care (at least one day)	6	Yes
Cardiovascular dysfunction or shock	7	Yes
Other aftercare (other diagnosis)	8	Yes
Early mechanical ventilation	9	Yes
Respiratory intubation and mechanical ventilation (secondary procedure)	10	Yes
Shock	11	Yes
Late mechanical ventilation	12	Yes
Insurance	13	Yes
Hepatic dysfunction	14	Yes
Other liver diseases (other diagnosis)	15	Yes
Coma, stupor, and brain damage (other diagnosis)	16	Yes
Location or teaching status of hospital	*17*	*No*
Bacterial infection, unspecified site (other diagnosis)	18	Yes
Race	19	Yes
Urinary tract infections (other diagnosis)	20	Yes
Pneumonia (except that caused by tuberculosis or sexually transmitted disease; other diagnosis)	21	Yes
Other gastrointestinal disorders (other diagnosis)	22	Yes
Joint disorders and dislocations, trauma-related (secondary diagnosis)	23	Yes
Acute and unspecified renal failure (other diagnosis)	24	Yes
Residual codes, unclassified (other diagnosis)	25	Yes
Aspiration pneumonitis and food or vomitus (other diagnosis)	26	Yes
Secondary malignancies (other diagnosis)	27	Yes
Anemia	28	Yes
Renal dysfunction	29	Yes
Other nervous system disorders (other diagnosis)	30	Yes
Other nutritional, endocrine, and metabolic disorders (other diagnosis)	31	Yes
Coagulation and hemorrhagic disorders (other diagnosis)	32	Yes
Other injuries and conditions because of external causes (other diagnosis)	33	Yes
Cardiac dysrhythmias (other diagnosis)	34	Yes
Insertion, replacement, or removal of extracranial ventricular shunt (primary procedure)	35	Yes
CNS^b^ dysfunction	36	Yes
Sex	37	Yes
Hemodialysis	38	Yes
Diabetes	39	Yes
Septicemia (except in labor; other diagnosis)	40	Yes
Nutritional deficiencies (other diagnosis)	41	Yes
Hypertension	42	Yes
Administrative or social admission (other diagnosis)	43	Yes
Allergic reactions (other diagnosis)	44	No
Pleurisy, pneumothorax, and pulmonary collapse (other diagnosis)	45	No
Metastatic cancer	46	Yes
Weight loss	47	Yes
Deficiency and other anemia (other diagnosis)	48	No
Delirium, dementia, and amnestic and other cognitive disorders (other diagnosis)	49	No
Coronary atherosclerosis and other heart disease (other)	50	No

^a^ICU: intensive care unit.

^b^CNS: central nervous system.

## Discussion

### Principal Findings

In this study, we applied 5 ML algorithms (LASSO, random forest, xgboost, deep neural network, and Super Learner) using variables from a national administrative database to predict in-hospital mortality in a sepsis cohort identified using the previously validated Martin implementation. The AUROCs of the ML models were in the excellent range (95% CI 0.877-0.895), supporting our ML models’ superior ability to discriminate mortality of patients with sepsis compared with the reference logistic regression model (95% CI 0.783-0.788). The ML models also showed superior AUC-PR measures (95% CI 0.636-0.681) compared with the reference logistic regression model (0.442). Among them, the models based on deep neural networks and xgboost outperformed the others in predicting sepsis mortality. To our knowledge, this is the first study to apply advanced ML models to predict sepsis mortality based on an administrative database.

It is important to distinguish between the 2 complementary uses of sepsis mortality risk prediction as they are distinct in their design and overall goals. EMR-integrated sepsis mortality prediction models are designed for use at the point of care to risk-stratify patients for clinical decision-making in the intensive care unit or emergency department [9-19]. However, unless proprietary systems are purchased, legal, technical, and financial barriers make it nearly impossible to extract the necessary clinical data from different EMR systems to assess performance across hospitals and states. In contrast, sepsis mortality prediction models based on administrative claims databases, which are available nationally, are designed to compare expected and actual sepsis mortality [20-24]. The latter was the focus of this study.

To date, traditional regression analyses have been applied to administrative data sources. Logistic regression of data ranging from single-center databases to regional and national databases has been used to predict sepsis mortality based on administrative data. Lagu et al [20] achieved an AUC of 0.78, Ford et al [21] achieved an AUC of 0.838, König et al [22] achieved an AUC >0.8, Schwarzkopf et al [24] achieved an AUC of 0.74, and Rhee et al [23] achieved an AUC of 0.776. In contrast to our approach, the aforementioned studies largely used traditional statistical models and did not use a validated Martin or Angus implementations approach to identify patients with sepsis. Moreover, they did not use the national inpatient database of the United States, which is the largest data set of US hospitalized patients. Recently, ML models have been applied to predicting sepsis mortality. Although some of them showed excellent performance, most of them were designed for point-of-care clinical application using the local EMR. In 2 previous studies using support vector machines, Ribas et al [12] achieved an AUC of 0.80, and Tsoukalas et al [13] obtained an AUC of 0.61. Taylor et al [14] used 500 clinical variables with a random forest model, which resulted in an AUC of 0.86. The study by Perng et al [15] used a support vector machine, k-nearest neighbor, random forest, and softmax with different extraction methods and achieved an AUC of 0.94. Kwon and Baek [16] used gradient boosting and random forests, achieving an AUC of 0.86. These ML algorithms were based on the local EMR database and may not be generalizable to other hospitals because of the case mix. By contrast, our ML models were based on a national administrative database with maximal generalizability [41].

As sepsis represents a major driver of cost and health care burden in the United States [4], improvement in sepsis care quality has been an important challenge. Considering the heterogeneous nature of sepsis, the calculation of sepsis risk-standardized mortality rates (RSMRs) is of great importance in measuring sepsis care quality across hospitals. Few relevant studies have been conducted based on a nationwide administrative database [23], and there remains much room for improved accuracy. Although hospital 30-day RSMRs for acute myocardial infarction, heart failure, and pneumonia have been reported by the Centers for Medicare and Medicaid Services [42], RSMRs for sepsis have not been well-characterized. Calculation of RSMR is important as identification of gaps between a facility’s RSMR and those of the state or nation’s highest-performing hospitals can lead hospital administrators, government policy makers, and other stakeholders to identify differences in practice and take action to improve sepsis care quality [43,44]. Disclosing discrepancies in RSMR also serves to reduce the asymmetry of information between consumers and health care providers and may spur market forces toward a more efficient allocation and distribution of health care resources to improve care [45]. To calculate RSMR across hospitals, our ML models were the first step in developing accurate models.

We believe that our mortality prediction model is an important tool that can be applied in health care research, quality improvement, and health policy making. However, our results should be interpreted with several limitations. First, any variation in the quality of coding in administrative data might affect the reliability of our study, including payment-related incentives for coding, over- or undercoding of conditions or risk factors, inconsistencies in coding practices between hospitals, and new technologies applied in sepsis care [46-48]. Second, the Martin implementation with which we extracted the sepsis cohort has been criticized for the less stringent use of septicemia and the omission of immunologic and coagulopathic organ dysfunction [30]. Third, we used in-hospital mortality as an outcome in our study and excluded patients who were transferred between hospitals. Consequently, those transferred against medical advice or to short-term hospitals were not counted. Whether this focus on in-hospital mortality could be biased by the hospital discharge policy warrants further investigation [49]. Fourth, some sepsis-related local characteristics such as local disease prevalence cannot be captured in a nationwide claim-based database. Thus, these variables could not be modeled and might influence our comparison results. Fifth, despite the excellent performance of the ML models, they suffer from varying degrees of explainability issues, and the inferences about variables (especially those that are clinically modifiable) tend to be more challenging [50]. Sixth, our model cannot be continuously updated because of the recent policy change of the NIS to eliminate state and hospital identifiers. As there is a time lag of >6 years, further research is needed to refine and update our ML models. However, the results of our training and validating analyses suggest that the accuracy of our model may not be significantly affected by time. Seventh, despite strong discrimination and performance, the data set used in this study was highly imbalanced, consisting of many more surviving patients. For future studies, one should consider down-sampling the survivor group to have a balanced data set before training the models and comparing the model performance. Eighth, although the non-ML logistic regression model using the same set of features as the ML models suffered from a statistically lower (*P*<.001) AUROC of 0.857 (95% CI 0.855-0.859), as documented in Table 8, some clinicians may prefer to use a model with easier interpretability, a drawback of the multilayered deep neural networks [51]. Ninth, the training and testing cohorts were split by year in this study to better capture the cyclic seasonal change in infection. The randomness of the training and testing cohort splitting could be compromised.

Nevertheless, our study has multiple strengths. First, we demonstrated the strength of ML models in predicting sepsis mortality in an administrative database. Second, the data we used were from a sepsis cohort extracted using a validated approach from the NIS database, which is a large, standardized, nationwide database representative of US community hospitals. Third, the variables used in our models are easily accessible across different hospitals, thus having great generalizability. Fourth, our large sample size enabled our ML models to discover complex multi-way interactions and nonlinear relationships between the predictors and outcomes, prompting further investigations for other clinical researchers. Fifth, to increase the reproducibility and usability of this research on sepsis care and mortality, we also generated a web-based application that will allow peer investigators to obtain predicted 30-day sepsis mortality calculations.

### Conclusions

In conclusion, our study demonstrates the value of ML models in predicting sepsis mortality in an administrative database as they are able to achieve higher discrimination and calibration. Knowledge of these ML models paves the way for the development of more accurate models to compare RSMRs across hospitals and geographic regions. This represents the first study to use an ML approach to improve the prediction of sepsis mortality in the NIS.

## Appendix

Multimedia Appendix 1 NIS_Sepsis_Main_Function.

Multimedia Appendix 2 Introductory video of our sepsis mortality prediction web application.

Multimedia Appendix 3 H2O ensemble Linux operating system.

Multimedia Appendix 4 H2O ensemble Windows operating system.

Multimedia Appendix 5 Coding book.

## Abbreviations

AUC: area under the curve
AUC-PR: area under the precision-recall curve
AUROC: area under the receiver operating characteristic curve
EMR: electronic medical record
LASSO: least absolute shrinkage and selection operator
ML: machine learning
NIS: National Inpatient Sample
RSMR: risk-standardized mortality rate
SHAP: Shapley Additive Explanations

## References

[1] Rhee C, Jones TM, Hamad Y, Pande A, Varon J, O'Brien C, Anderson DJ, Warren DK, Dantes RB, Epstein L, Klompas M, Centers for Disease Control and Prevention (CDC) Prevention Epicenters Program (2019). Prevalence, underlying causes, and preventability of sepsis-associated mortality in US acute care hospitals. JAMA Netw Open.

[2] Wang HE, Jones AR, Donnelly JP (2017). Revised national estimates of emergency department visits for sepsis in the United States. Crit Care Med.

[3] Moore JX, Donnelly JP, Griffin R, Howard G, Safford MM, Wang HE (2016). Defining sepsis mortality clusters in the United States. Crit Care Med.

[4] Paoli CJ, Reynolds MA, Sinha M, Gitlin M, Crouser E (2018). Epidemiology and costs of sepsis in the United States-an analysis based on timing of diagnosis and severity level. Crit Care Med.

[5] Bergmann S, Tran M, Robison K, Fanning C, Sedani S, Ready J, Conklin K, Tamondong-Lachica D, Paculdo D, Peabody J (2019). Standardising hospitalist practice in sepsis and COPD care. BMJ Qual Saf.

[6] Lemeshow S, Teres D, Klar J, Avrunin JS, Gehlbach SH, Rapoport J (1993). Mortality Probability Models (MPM II) based on an international cohort of intensive care unit patients. JAMA.

[7] Ferreira FL, Bota DP, Bross A, Mélot C, Vincent JL (2001). Serial evaluation of the SOFA score to predict outcome in critically ill patients. JAMA.

[8] Knaus WA (2002). APACHE 1978-2001: the development of a quality assurance system based on prognosis: milestones and personal reflections. Arch Surg.

[9] Sivayoham N, Rhodes A, Cecconi M (2014). The MISSED score, a new scoring system to predict mortality in severe sepsis in the emergency department: a derivation and validation study. Eur J Emerg Med.

[10] Granja C, Póvoa P, Lobo C, Teixeira-Pinto A, Carneiro A, Costa-Pereira A (2013). The predisposition, infection, response and organ failure (Piro) sepsis classification system: results of hospital mortality using a novel concept and methodological approach. PLoS One.

[11] Osborn TM, Phillips G, Lemeshow S, Townsend S, Schorr CA, Levy MM, Dellinger RP (2014). Sepsis severity score: an internationally derived scoring system from the surviving sepsis campaign database*. Crit Care Med.

[12] Ribas V, López JC, Ruiz-Sanmartin A, Ruiz-Rodríguez JC, Rello J, Wojdel A, Vellido A (2011). Severe sepsis mortality prediction with relevance vector machines. Annu Int Conf IEEE Eng Med Biol Soc.

[13] Tsoukalas A, Albertson T, Tagkopoulos I (2015). From data to optimal decision making: a data-driven, probabilistic machine learning approach to decision support for patients with sepsis. JMIR Med Inform.

[14] Taylor RA, Pare JR, Venkatesh AK, Mowafi H, Melnick ER, Fleischman W, Hall MK (2016). Prediction of in-hospital mortality in emergency department patients with sepsis: a local big data-driven, machine learning approach. Acad Emerg Med.

[15] Perng JW, Kao IH, Kung CT, Hung SC, Lai YH, Su CM (2019). Mortality prediction of septic patients in the emergency department based on machine learning. J Clin Med.

[16] Kwon YS, Baek MS (2020). Development and validation of a quick sepsis-related organ failure assessment-based machine-learning model for mortality prediction in patients with suspected infection in the emergency department. J Clin Med.

[17] Joshi M, Ashrafian H, Arora S, Khan S, Cooke G, Darzi A (2019). Digital alerting and outcomes in patients with sepsis: systematic review and meta-analysis. J Med Internet Res.

[18] Sandhu S, Lin AL, Brajer N, Sperling J, Ratliff W, Bedoya AD, Balu S, O'Brien C, Sendak MP (2020). Integrating a machine learning system into clinical workflows: qualitative study. J Med Internet Res.

[19] Muralitharan S, Nelson W, Di S, McGillion M, Devereaux P, Barr NG, Petch J (2021). Machine learning-based early warning systems for clinical deterioration: systematic scoping review. J Med Internet Res.

[20] Lagu T, Lindenauer PK, Rothberg MB, Nathanson BH, Pekow PS, Steingrub JS, Higgins TL (2011). Development and validation of a model that uses enhanced administrative data to predict mortality in patients with sepsis. Crit Care Med.

[21] Ford DW, Goodwin AJ, Simpson AN, Johnson E, Nadig N, Simpson KN (2016). A severe sepsis mortality prediction model and score for use with administrative data. Crit Care Med.

[22] König V, Kolzter O, Albuszies G, Thölen F (2018). Einflussgrößen auf die krankenhaussterblichkeit bei patienten mit sepsis – entwicklung eines risikoadjustierten modells auf basis der leistungsdaten deutscher krankenhäuser. Z Evid Fortbild Qual Gesundhwes.

[23] Rhee C, Wang R, Song Y, Zhang Z, Kadri SS, Septimus EJ, Fram D, Jin R, Poland RE, Hickok J, Sands K, Klompas M (2019). Risk adjustment for sepsis mortality to facilitate hospital comparisons using centers for disease control and prevention's adult sepsis event criteria and routine electronic clinical data. Crit Care Explor.

[24] Schwarzkopf D, Fleischmann-Struzek C, Rüddel H, Reinhart K, Thomas-Rüddel DO (2018). A risk-model for hospital mortality among patients with severe sepsis or septic shock based on German national administrative claims data. PLoS One.

[25] Doupe P, Faghmous J, Basu S (2019). Machine learning for health services researchers. Value Health.

[26] Song X, Mitnitski A, Cox J, Rockwood K (2004). Comparison of machine learning techniques with classical statistical models in predicting health outcomes. Stud Health Technol Inform.

[27] (2011). Introduction to the HCUP nationwide inpatient sample (NIS). Healthcare Cost and Utilization Project (HCUP).

[28] (2012). Introduction to the HCUP national inpatient sample (NIS). Healthcare Cost and Utilization Project (HCUP).

[29] Martin GS, Mannino DM, Eaton S, Moss M (2003). The epidemiology of sepsis in the United States from 1979 through 2000. N Engl J Med.

[30] Iwashyna TJ, Odden A, Rohde J, Bonham C, Kuhn L, Malani P, Chen L, Flanders S (2014). Identifying patients with severe sepsis using administrative claims: patient-level validation of the angus implementation of the international consensus conference definition of severe sepsis. Med Care.

[31] Breiman L (2001). Random forests. Mach Learn.

[32] Lundberg S, Lee SI A unified approach to interpreting model predictions. arXiv..

[33] van der Laan MJ, Polley EC, Hubbard AE (2007). Super learner. Stat Appl Genet Mol Biol.

[34] Friedman J, Hastie T, Tibshirani R (2010). Regularization paths for generalized linear models via coordinate descent. J Stat Softw.

[35] Churpek MM, Yuen TC, Winslow C, Meltzer DO, Kattan MW, Edelson DP (2016). Multicenter comparison of machine learning methods and conventional regression for predicting clinical deterioration on the wards. Crit Care Med.

[36] Winterstein AG, Choi Y, Meissner HC (2018). Association of age with risk of hospitalization for respiratory syncytial virus in preterm infants with chronic lung disease. JAMA Pediatr.

[37] Breiman L (1996). Bagging predictors. Mach Learn.

[38] Friedman JH (2001). Greedy function approximation: a gradient boosting machine. Ann Statist.

[39] Abiodun OI, Jantan A, Omolara AE, Dada KV, Mohamed NA, Arshad H (2018). State-of-the-art in artificial neural network applications: a survey. Heliyon.

[40] Sepsis Mortality Prediction.

[41] Gandhi S, Salmon JW, Kong SX, Zhao SZ (1999). Administrative databases and outcomes assessment: an overview of issues and potential utility. J Manag Care Pharm.

[42] (2021). Outcome measures. Centers for Medicare & Medicaid Services.

[43] Berta P, Martini G, Moscone F, Vittadini G (2016). The association between asymmetric information, hospital competition and quality of healthcare: evidence from Italy. J R Stat Soc Ser A Stat Soc.

[44] Desai NR, Ott LS, George EJ, Xu X, Kim N, Zhou S, Hsieh A, Nuti SV, Lin Z, Bernheim SM, Krumholz HM (2018). Variation in and hospital characteristics associated with the value of care for Medicare beneficiaries with acute myocardial infarction, heart failure, and pneumonia. JAMA Netw Open.

[45] Angst C, Agarwal R, Gao GG, Khuntia J, McCullough JS (2014). Information technology and voluntary quality disclosure by hospitals. Decis Support Syst.

[46] Iezzoni LI (1997). Assessing quality using administrative data. Ann Intern Med.

[47] Farmer SA, Black B, Bonow RO (2013). Tension between quality measurement, public quality reporting, and pay for performance. JAMA.

[48] Hashimoto RE, Brodt ED, Skelly AC, Dettori JR (2014). Administrative database studies: goldmine or goose chase?. Evid Based Spine Care J.

[49] Vasilevskis EE, Kuzniewicz MW, Dean ML, Clay T, Vittinghoff E, Rennie DJ, Dudley RA (2009). Relationship between discharge practices and intensive care unit in-hospital mortality performance: evidence of a discharge bias. Med Care.

[50] London AJ (2019). Artificial intelligence and black-box medical decisions: accuracy versus explainability. Hastings Cent Rep.

[51] Christodoulou E, Ma J, Collins GS, Steyerberg EW, Verbakel JY, Van Calster B (2019). A systematic review shows no performance benefit of machine learning over logistic regression for clinical prediction models. J Clin Epidemiol.

