# Supplementary material for: Predicting Sepsis Mortality in a Population-Based National Database: Machine Learning Approach
Source: J Med Internet Res. 2022 Apr 13;24(4):e29982. doi: 10.2196/29982 (PMC9047761; doi:10.2196/29982)
Supplement: Multimedia Appendix 1 [file jmir_v24i4e29982_app1.pdf]

```
############################################
# Install Dependencies
############################################

# install packages required for the models
libraries <- c("glmnet", "rpart", "party", "partykit", "gbm", "SuperLearner", "fastDummies", "tensorflow",
"ROCR", "tableone",
               "ranger", "rms", "pROC", "keras", "data.table", "usdm","xgboost", "reportROC",
"SHAPforxgboost", "parallel")

lapply(libraries, require, character.only = TRUE)



############################################
# Main Class
############################################

main <- function(){

  # change wd
  setwd(file_wd)

  # read the file
  sepsis_df <- process_df("ana4_3.txt")

  # fit logistic model
  roc_logistic <- fit_logit_model(sepsis_df)
```

```
# fit lasso  model
pred_lasso_prob <- fit_lasso(sepsis_df)

# Random forest - best paraemters after sensitivity analysis
pred_RF_Final <- fit_random_forest(train_outcome, sepsis_train, 400, 500)

# Xgboost - best set of parameters after parameter tuning
boosted_pred <- fit_xgboost(sepsis_train, sepsis_validation, train_outcome, 0.15, 10, 400)

# NNet - architecture/parameter defined after sensitiviry analysis
nnet_predict <- fit_nnet_model(sepsis_train, sepsis_validation, 6)

# super learner
super_predict <- fit_super_learner(sepsis_train, train_outcome, sepsis_validation)

# Calibration Curve Example
lasso_val <- val.prob(p= pred_lasso_prob, y= test_outcome, m=20, cex=.5, smooth = T,
                      xlab ="Predicted Probability", ylab="Actual Probability")

# Brier score example
lasso_val <- val.prob(pred_lasso_prob, y = test_outcome, g = 10, smooth = T, logistic.cal =F,
                      legendloc=F, statloc = F, pl=F)
lasso_val["Brier"]

# ROC Curve Example
plot(roc_logistic, col = 'orange', lwd = 3)

# Precision Curve Example
lasso_pred <- prediction(pred_lasso_prob, test_outcome)
```

```
  lasso_perf <- performance(lasso_pred,"prec","rec")
  plot(lasso_perf, avg= "threshold", lwd= 3,col = 'orange',main= "Lasso - Precision Recall Curve", 
spread.scale = 2)

  # fitting ROC object and perform pairwise ROC comparsion
  roc_logistic <- roc(test_outcome, pred_prob_logreg, ci=TRUE, of="auc")
  roc_lasso <- roc(test_outcome, pred_lasso_prob, ci=TRUE, of="auc")
  roc_rf <- roc(test_outcome, RF_Final.pred, ci=TRUE, of="auc")
  roc_xgboost <- roc(test_outcome, boosted_pred, ci=TRUE, of="auc")
  roc_nnet <- roc(test_outcome, nnet_final, ci=TRUE, of="auc")

  roc.test(roc_logistic, roc_lasso) # returns p-value from DeLong's test

  # Descriptive Statistics example
  descriptive_var <- c("AGE", "FEMALE", "c14", "c15", "c16", "c17", "c20", "c13", "c12",
                    "c7", "c21", "c18", "c11", "DEPRESS", "c10", "DRUG", "CHRNLUNG",
                    "CHF", "HTN_C", "HYPOTHY", "LIVER", "LYMPH", "METS", "NEURO", "OBESE",
                    "PARA", "PERIVASC", "PSYCH", "PULMCIRC", "RENLFAIL", "TUMOR",
                    "WGHTLOSS", "c6", "c1", "c2", "c3", "c4", "c5", "c8", "c9",'ZIPInc_Qrtl','ARTH')

  cat_var <- c("FEMALE", "c14", "c15", "c16", "c17", "c20", "c13", "c12",
            "c7", "c21", "c18", "c11", "DEPRESS", "c10", "DRUG", "CHRNLUNG",
            "CHF", "HTN_C", "HYPOTHY", "LIVER", "LYMPH", "METS", "NEURO", "OBESE",
            "PARA", "PERIVASC", "PSYCH", "PULMCIRC", "RENLFAIL", "TUMOR",
            "WGHTLOSS", "c6", "c1", "c2", "c3", "c4", "c5", "c8", "c9",'ZIPInc_Qrtl','ARTH')

  CreateTableOne(vars = descriptive_var, strata = "DIED" , data = sepsis_df,factorVars = cat_var)

}
```

```
############################################
# Data Pre-Processing
############################################

process_df <- function(df_file){

  sepsis_df <- as.data.frame(fread(df_file))

  ## remove biasing variables
  delete <- c("prccs_cat_p232","prccs_cat_p233","prccs_cat_s232","prccs_cat_s233",
              "dxccs_cat_s107", "dxccs_cat_s131", "dxccs_cat_p107", "dxccs_cat_p131",
              "dxccs_cat_o107", "dxccs_cat_o131")

  sepsis_df <- sepsis_df[,!(names(sepsis_df) %in% delete)]

  # remove any rows without NA after converting to integer (n = 1015514)
  sepsis_df <- as.data.frame(lapply(sepsis_df, as.integer))
  sepsis_df <<- sepsis_df[complete.cases(sepsis_df), ]

  #*----------------- Train/Test Split
  ## global variables
  # train data (2010 - 2013) (n = 704,246)
  sepsis_train <<- sepsis_df[sepsis_df$YEAR %in% c(2010, 2011, 2012, 2013),]
  sepsis_train <<- sepsis_train[, -6] # remove 'year'
  train_outcome <<- sepsis_train$DIED
  sepsis_train <<- sepsis_train[, -2] # remove 'DIED'

  # validation data (2014) (n = 219,513)
```

```
  sepsis_validation <<- sepsis_df[sepsis_df$YEAR %in% c(2014),]
  sepsis_validation <<- sepsis_validation[, -6]
  test_outcome <<- sepsis_validation$DIED
  sepsis_validation <<- sepsis_validation[, -2]

  return(sepsis_df)
}

############################################
# METHOD 1- REGULAR LOGISTIC REGRESSION
############################################

fit_logit_model <- function(sepsis_df){

  # age stratification
  logistic_df <- sepsis_df
  logistic_df$AGE <- ifelse(logistic_df$AGE< 40, 0,
                  ifelse(39 < logistic_df$AGE & logistic_df$AGE < 50, 8,
                  ifelse(49 < logistic_df$AGE & logistic_df$AGE < 60, 10,
                  ifelse(59 < logistic_df$AGE & logistic_df$AGE < 70, 12,
                  ifelse(69 < logistic_df$AGE & logistic_df$AGE < 80, 15,
                  ifelse(79 < logistic_df$AGE & logistic_df$AGE < 90, 18, 23))))))

  # gender stratification
  logistic_df$FEMALE <- ifelse(logistic_df$FEMALE == 1, 6, 0)

  # ethnicity
  logistic_df$c16 <- ifelse(logistic_df$c16 == 1, 0, 0) # white
  logistic_df$c17 <- ifelse(logistic_df$c17 == 1, 0, 0) # others
```

```
logistic_df$c14 <- ifelse(logistic_df$c14 == 1, 6, 0) # black
logistic_df$c15 <- ifelse(logistic_df$c15 == 1, 6, 0) # hispanic

# ventilation
logistic_df$c13 <- ifelse(logistic_df$c13 == 1, 20, 0)
logistic_df$c12 <- ifelse(logistic_df$c12 == 1, 23, 0)

# shock, hemodialysis, ICU
logistic_df$c7 <- ifelse(logistic_df$c7 == 1, 12, 0)
logistic_df$c21 <- ifelse(logistic_df$c21 == 1, 10, 0)
logistic_df$c18 <- ifelse(logistic_df$c18 == 1, 2, 0)

# comorbidity
logistic_df$c11 <- ifelse(logistic_df$c11 == 1, 1, 0)
logistic_df$DEPRESS <- ifelse(logistic_df$DEPRESS == 1, 3, 0)
logistic_df$c10 <- ifelse(logistic_df$c10 == 1, 4, 0)
logistic_df$DRUG <- ifelse(logistic_df$DRUG == 1, 1, 0)
logistic_df$CHRNLUNG <- ifelse(logistic_df$CHRNLUNG == 1, 4, 0)
logistic_df$CHF <- ifelse(logistic_df$CHF == 1, 5, 0)
logistic_df$HTN_C <- ifelse(logistic_df$HTN_C == 1, 3, 0)

# train/validation split
logistic_train <<- logistic_df[logistic_df$YEAR %in% c(2010, 2011, 2012, 2013),]
logistic_train <<- logistic_train[, -6] # remove 'year'
train_outcome <- logistic_train$DIED
logistic_train <<- logistic_train[, -2] # remove 'DIED'

# validation data (2014) (n = 219,513)
logistic_validation <<- logistic_df[logistic_df$YEAR %in% c(2014),]
```

```
  logistic_validation <<- logistic_validation[, -6]
  test_outcome <- logistic_validation$DIED
  logistic_validation <<- logistic_validation[, -2]

  logit_variables <- c('AGE', 'FEMALE', 'c16','c17','c14','c15','c13','c12','c7',
                       'c21','c18','c11','DEPRESS','c10', 'DRUG', 'CHRNLUNG', 'CHF', 'HTN_C')

  # select only those that we need for the model
  logistic_train <<- logistic_train[,logit_variables]
  logistic_validation <<- logistic_validation[,logit_variables]

  # traditional logistic regression
  logistic_train <- sepsis_train
  logreg <- glm(as.matrix(train_outcome) ~ ., data = logistic_train, family=binomial)

  #Make predictions using the test data set (overfitting)
  pred_prob_logreg     <- predict(logreg, newdata=logistic_validation, type     = "response")
  logistic_val <- val.prob(p= pred_prob_logreg, y= test_outcome, g=10, smooth=T,
                           logistic.cal = F, legendloc= F, statloc = F, pl=F)

  # print performance stats
  print(reportROC(test_outcome, pred_prob_logreg))

  # logistic ROC object
  roc_logistic <- roc(test_outcome, pred_prob_logreg, ci=TRUE, of="auc")

  return(pred_prob_logreg)
}
```

```
############################################
# METHOD 2- LASSO
############################################

fit_lasso <- function(sepsis_df){

  # categorize non-continuous variables
  lasso_df <- sepsis_df
  lasso_df[,c(2:5,7:24,26:1328)] <- lapply(lasso_df[,c(2:5,7:23,25:1328)], as.factor)

  # train/validation split
  lasso_train <- lasso_df[lasso_df$YEAR %in% c(2010, 2011, 2012, 2013),]
  lasso_validaiton <- lasso_df[lasso_df$YEAR %in% c(2014),]

  # Create matrices of training set
  y <- lasso_train %>% dplyr::select(DIED) %>%
    data.matrix() -1
  x <- lasso_train %>% dplyr::select(-c(DIED,YEAR)) %>%
    data.matrix(rownames.force = T)

  # Create matrices of test set
  y_test <- lasso_validaiton %>% dplyr::select(DIED)  %>% data.matrix() -1
  x_test <- lasso_validaiton %>% dplyr::select(-c(DIED,YEAR)) %>% data.matrix(rownames.force = T)

  # Sanity check -- no NA alllowed
  sum(is.na(c(x,y,x_test,y_test)))

  # Fit Lasso in training set with cross validation to identify the best lambda
  set.seed(1)
```

```
  fit_lasso_cv <- cv.glmnet(x, y,
                         family = "binomial",
                         type.measure = "mse", nfolds = 10,
                         standardize = TRUE)

  # Save this module for the future
  saveRDS(fit_lasso_cv, 'Lasso.rds')
  fit_lasso_cv <- readRDS('Lasso.rds')

  # Prediction
  pred_lasso_prob <- predict(logreg, newx = x_test,
                          s = "lambda.min", type="response") %>% as.vector()

  # all statistics
  print(reportROC(as.factor(y_test), pred_lasso_prob))

  return(pred_lasso_prob)

}

############################################
## METHOD 3- Random Forest
############################################

fit_random_forest <- function(train_outcome, sepsis_train, num_tree, num_split){

  # ranger -- faster/more implementation of random forest (C++)
  set.seed(555)
```

```
RF_Final <- ranger(as.factor(train_outcome) ~ ., data=sepsis_train,
                   importance = "impurity",
                   num.trees = num_tree, # number of trees
                   mtry = num_split, # number of variables to split at in each node
                   probability = TRUE)


# Save this module for the future
saveRDS(RF_Final, 'RF_Final')
RF_Final <- readRDS('RF_Final')

# variable of importance
v <- as.vector(RF_Final$variable.importance)
w <- as.vector((colnames(sepsis_train)))
DF <- as.data.frame(cbind(w, v))
DF$v <- as.numeric(as.character(DF$v))

DF_50 <- head(arrange(DF,desc(v),w),50) # 50 most important variables


pdf("var_importance.pdf")

ggplot(DF_50, aes(x = reorder(w,v), y = v))+
  geom_bar(stat="identity", position="dodge")+ coord_flip()+
  ylab("Variable Importance")+
  xlab("")+
  ggtitle("Top 50 Most Important Variables from Random Forest")+
  guides(fill=F)+
  scale_fill_gradient(low="red", high="blue") +
```

```
    theme_classic()+
    scale_x_discrete(expand = c(0, 0))

  dev.off()

  # prediction
  pred_RF_Final <<- predict(RF_Final, data = sepsis_validation, type = "response") #gives out a non-
numeric matrix
  RF_Final.pred <<- pred_RF_Final$predictions[,2] # only keep probability of outcome=1

  # prediction ROC
  RF_ROC <<- roc(test_outcome,RF_Final.pred, ci = TRUE, of = 'auc')

  # all statisstics
  print(reportROC(test_outcome, RF400_mtry50.pred))

  return(RF_Final.pred)
}

############################################
## METHOD 4 - xgboost
############################################

fit_xgboost <- function(sepsis_train, sepsis_validation, train_outcome, lr, depth, tree){

  # change frames to matrices
  set.seed(9991)
  sepsis_train_gbm <- xgb.DMatrix(data = as.matrix(sepsis_train), label = train_outcome)
  sepsis_validation_gbm <- xgb.DMatrix(data = as.matrix(sepsis_validation), label = test_outcome)
```

```
# xgboost is a parallelized version of gbm
boosted <- xgb.train(data=sepsis_train_gbm,
            params = list(object = "binary:logistic",
                    eta = lr, # learning rate -- lower eta ~ larger rounds
                    max_depth = depth, # max depth of tree
                    nthread = 10, # parallel processing
                    eval_metric = 'auc'),
            watchlist = list(test = sepsis_validation_gbm),
            nrounds = 400,
            early_stopping_rounds = 50,
            maximize = TRUE, # maximize AUC
            ntrees = tree, # use 100 tree to tune the parameter,
            nfolds = 10, # 10 fold validation
            distribution = 'bernoulli')

# Save this module for the future
saveRDS(boosted, 'boosted_final.rds')
boosted_final <- readRDS("boosted_final.rds")

# prediction
boosted_pred <<- predict(boosted,
              newdata = sepsis_validation_gbm,
              ntreelimit = boosted$bestInd)

# auc with 95% CI
gbm_auc <<- roc(test_outcome, boosted_pred, plot = TRUE, col = "blue", ci = TRUE)

# all statisstics
```

```
print(reportROC(test_outcome, boosted_pred))

# SHAP score
shap_values <- shap.prep(xgb_model = boosted_final, X_train = sepsis_train)
saveRDS(shap_values, 'xg_shap.rds')
shap_values <- readRDS("xg_shap.rds")
shap_values <- shap_values[,c('variable','mean_value')]
shap_values <- as.data.frame(shap_values[!duplicated(shap_values), ])

# export shapley plot
theme_set(theme_bw())
pdf('shap_plot.pdf')

ggplot(shap_values[order(-shap_values$mean_value),], aes(x = mean_value, y = variable))+
  geom_point(size = 3) +
  geom_segment(aes(x=0,
                xend=mean_value,
                yend=variable,
                y = variable)) +
  labs(title = 'Top 50 variables in xgboost by SHAP values') +
  xlab('mean SHAP value') +
  ylab('variable') +
  theme(plot.title = element_text(hjust = 0.5)) +
  scale_x_continuous(expand = c(0, 0), limits = c(0, 0.1))

dev.off()

# VIF score - multicolinearity
pdf('vif_score.pdf')
```

```
  vif_results <- usdm::vif(sepsis_df[,shap_values$variable])
  colnames(vif_results) <- c('Variable', 'VIF')

  ggplot(vif_results[order(vif_results$VIF),], aes(x = VIF, y = Variable))+
    geom_point(size = 3) +
    geom_segment(aes(x=0,
                     xend=VIF,
                     yend=Variable,
                     y = Variable)) +
    labs(title = 'VIF Scores of Top 50 variables by SHAP values') +
    xlab('VIF') +
    ylab('') +
    theme(plot.title = element_text(hjust = 0.5)) +
    scale_x_continuous(expand = c(0, 0), limits = c(0, 17))
  dev.off()

  return(boosted_pred)
}

############################################
# METHOD 5 - NeuralNetworks
############################################

fit_nnet_model <- function(sepsis_train, sepsis_validation, patience){

  # nnet objects
  set.seed(1010)
  neural_train <- sepsis_train
  neural_test <- sepsis_validation
```

```
# continuous variables normalization (z-score)

neural_train$AGE <- (neural_train$AGE - mean(neural_train$AGE))/sd(neural_train$AGE)

neural_train$SID29 <- (neural_train$SID29 - mean(neural_train$SID29))/sd(neural_train$SID29)

neural_test$AGE <- (neural_test$AGE - mean(neural_test$AGE))/sd(neural_test$AGE)

neural_test$SID29 <- (neural_test$SID29 - mean(neural_test$SID29))/sd(neural_test$SID29)


# categorical variable as dummy variables

neural_train <- fastDummies::dummy_cols(neural_train, select_columns = "c19", remove_first_dummy = TRUE)

neural_test <- fastDummies::dummy_cols(neural_test, select_columns = "c19", remove_first_dummy = TRUE)

neural_train <- fastDummies::dummy_cols(neural_train, select_columns = "c20", remove_first_dummy = TRUE)

neural_test <- fastDummies::dummy_cols(neural_test, select_columns = "c20", remove_first_dummy = TRUE)

neural_train <- fastDummies::dummy_cols(neural_train, select_columns = "RACE", remove_first_dummy = TRUE)

neural_test <- fastDummies::dummy_cols(neural_test, select_columns = "RACE", remove_first_dummy = TRUE)

neural_train <- fastDummies::dummy_cols(neural_train, select_columns = "ZIPInc_Qrtl", remove_first_dummy = TRUE)

neural_test <- fastDummies::dummy_cols(neural_test, select_columns = "ZIPInc_Qrtl", remove_first_dummy = TRUE)


# binary variables (-1,1 scale)

neural_train[, c(2:3,6:22,24:42,45:ncol(neural_train))][neural_train[, c(2:3,6:22,24:42,45:ncol(neural_train))] == 0] <- -1

neural_test[, c(2:3,6:22,24:42,45:ncol(neural_test))][neural_test[, c(2:3,6:22,24:42,45:ncol(neural_test))] == 0] <- -1


# NNet CANNOT handle missing values
```

```
train_Y = as.matrix(train_outcome) # Y outcome for train
neural_train = as.matrix(neural_train)
neural_train[is.na(neural_train)] <- 0

# keras feed-forwarding model
model <- keras_model_sequential() %>%

  # 4 deep layer model workd the best
  # dropout and regularization hurts the AUC and will not be used
  layer_dense(units = 32, activation = "relu", input_shape = ncol(neural_train)) %>%
  layer_batch_normalization() %>% # batch norm is enough to combat overiftting

  layer_dense(units = 16, activation = "relu") %>%
  layer_batch_normalization() %>%

  layer_dense(units = 4, activation = "relu") %>%
  layer_batch_normalization() %>%

  layer_dense(units = 1, activation = "sigmoid") %>% # sigmoid activation for binary classification

  compile(
    optimizer = "adam", # adam optimization
    loss = "binary_crossentropy", # binary cross entropy for binary classification loss
    metrics = c("AUC") # AUC to assess the model performacne
  )

# deep learning learning
learn <- model %>% fit(
  x = as.matrix(neural_train),
```

```
  y = train_Y,
  epochs = 35, # determined after validation loss stopped decreasing at epoch 25
  batch_size = 32, # 32 batch size for the CPU memory
  validation_split = .1, # 10-fold cross validation
  verbose = FALSE,
  callbacks = list( # stop epoch if not improving
    callback_early_stopping(patience = patience),
    callback_reduce_lr_on_plateau() # adjust learning rate
  )
)

# train & validation curve
pdf('final_tuned.pdf')
plot(learn, main = 'Sepsis Neural Network Train and Validation Performance')
dev.off()

# nnet can't handle missing valurs
test_Y = as.matrix(test_outcome) # Y outcome for train
neural_test[is.na(neural_test)] <- 0
neural_test =  as.matrix(neural_test)

# prediction
test_predict <<- model %>% predict(neural_test) # model evaludation on the test-set
saveRDS(test_predict, 'nnet_predict.rds')

nnet_auc <<- roc(test_Y, test_predict, plot = TRUE, col = "blue", ci = TRUE)
print(reportROC(test_Y, test_predict))

return(test_predict)
```

```
}


###########################################
# METHOD 6 - Super Learner (Test using SuperLeanrer package)
###########################################

fit_super_learner <- function(sepsis_train, train_outcome, sepsis_validation){

  # gbm matrix
  x_mat <- model.matrix(~.,data=sepsis_train)
  x_mat <- x_mat[,-1] # remove the intercept
  x_mat <- as.data.frame(x_mat)

  # nonparallel
  superboost <- SuperLearner(train_outcome,x_mat,family=binomial(),SL.library = "SL.xgboost")
  saveRDS(superboost, 'superboost.rds')

  ## parallel version
  cl <- makeCluster(detectCores()-2)

  clusterExport(cl, varlist =
                c('SuperLearner',
                  'x_mat' ,'train_outcome',
                  'family','SL.library'))


  clusterSetRNGStream(cl, iseed=135)
```

```
  clusterEvalQ(cl, {
   library(SuperLearner);
   library(xgboost)
  })

  # Run training session in parallel
  clusterEvalQ(cl, {
   super_boost <- SuperLearner(train_outcome,x_mat,
                              family=binomial(), SL.library="SL.xgboost"
   );saveRDS(super_boost ,'superboost.rds')
  })



  # prediction using these samples
  super_predict <<- predict(super_xgboost,sepsis_validation,type='response')

  return(super_predict)
}
```
