# Supplementary material for: Predicting Sepsis Mortality in a Population-Based National Database: Machine Learning Approach
Source: J Med Internet Res. 2022 Apr 13;24(4):e29982. doi: 10.2196/29982 (PMC9047761; doi:10.2196/29982)
Supplement: Multimedia Appendix 3 [file jmir_v24i4e29982_app3.pdf]

```
#setwd("/Users/hojoshua/Desktop/Monica")
setwd("/root/H2O-1")

###########################################
# Install and import Dependencies
###########################################
# install packages required for the models
libraries <- c("glmnet", "rpart", "party", "partykit", "gbm", "SuperLearner", "fastDummies", "tensorflow",
              "ranger", "rms", "pROC", "keras", "data.table", "xgboost", "reportROC", "SHAPforxgboost",
"parallel")

lapply(libraries, require, character.only = TRUE)

#New package
#library(microbenchmark)
library(h2o)
h2o.init(nthreads=detectCores()-1, max_mem_size="40G")
h2o.removeAll() ## clean slate - just in case the cluster was already running

###################################################
#  Import a binary outcome train/test set into H2O#
##################################################

# Import a sample binary outcome train/test set into H2O
train <- h2o.importFile("/root/H2O-1/sepsis_train_small.txt") # use abs path
test <- h2o.importFile("/root/H2O-1/sepsis_validation_small.txt") # use abs path

y <- "DIED"
x <- setdiff(names(train), y)
```

```
train[,y] <- as.factor(train[,y])
test[,y] <- as.factor(test[,y])

nfolds <- 10



####################################################
#  rf model #
###################################################
s2 <- Sys.time()

my_rf <- h2o.randomForest(x = x,
                    y = y,
                    training_frame = train,
                    ntrees = 400,
                    mtries=500,
                    nfolds=10, #this has to be the same, by JH
                    keep_cross_validation_predictions = TRUE,
                    seed = 1) #this has to be the same, by JH
t2 <- Sys.time()-s2
# Save this module for the future
model_my_rf <- h2o.saveModel(object = my_rf, path = getwd(), force = TRUE)
fwrite(as.list(t2),"t2.txt")

####################################################
#  XGB model   not available on windows OS#
###################################################
s3 <- Sys.time()
```

```
XGB <- h2o.xgboost(x = x,
          y = y,
          training_frame = train,
          eta = 0.15,
          max_depth = 10,
          #nthread = 10,
          stopping_metric = "AUC",
          stopping_rounds = 50,
          ntrees = 400,
          nfolds=10, #this has to be the same, by JH
          distribution = 'bernoulli',
                                  keep_cross_validation_predictions = TRUE, #need this, by JH
          seed=1) #this has to be the same, by JH
t3 <- Sys.time()-s3
# Save this module for the future
model_XGB <- h2o.saveModel(object = XGB, path = getwd(), force = TRUE)
fwrite(as.list(t3),"t3.txt")

##################################################
#  ensemble model   not available on windows OS#
##################################################
s4 <- Sys.time()
ensemble <- h2o.stackedEnsemble(x = x,
                y = y,
                training_frame = train,
                base_models = list(my_rf,XGB))
t4 <- Sys.time()-s4
# Save this module for the future
model_ensemble <- h2o.saveModel(object = ensemble, path = getwd(), force = TRUE)
```

```
fwrite(as.list(t4),"t4.txt")


####################################################
#  Performance#
###################################################

perf <- h2o.performance(ensemble, newdata = test)
ensemble_auc_test <- h2o.auc(perf)



perf_rf_test <- h2o.performance(my_rf, newdata = test)
perf_XGB_test <- h2o.performance(XGB, newdata = test)

baselearner_best_auc_test <- max(h2o.auc(perf_rf_test),
                              h2o.auc(perf_XGB_test))



print(sprintf("Best Base-learner Test AUC: %s", baselearner_best_auc_test))
print(sprintf("Ensemble Test AUC: %s", ensemble_auc_test))



# [1] "Best Base-learner Test AUC:  0.76979821502548"
# [1] "Ensemble Test AUC:  0.773501212640419"
```
