# Supplementary material for: Predicting Sepsis Mortality in a Population-Based National Database: Machine Learning Approach
Source: J Med Internet Res. 2022 Apr 13;24(4):e29982. doi: 10.2196/29982 (PMC9047761; doi:10.2196/29982)
Supplement: Multimedia Appendix 4 [file jmir_v24i4e29982_app4.pdf]

```r
setwd("F:\\NIS_SEPSIS\\test\\")


############################################
# Install and import Dependencies
############################################
# install packages required for the models
libraries <- c("glmnet", "rpart", "party", "partykit", "gbm", "SuperLearner", "fastDummies", "tensorflow",
               "ranger", "rms", "pROC", "keras", "data.table", "xgboost", "reportROC", "SHAPforxgboost",
"parallel")


lapply(libraries, require, character.only = TRUE)


#New package
library(microbenchmark)
library(h2o)
h2o.init(nthreads=detectCores()-1, max_mem_size="40G")
h2o.removeAll() ## clean slate - just in case the cluster was already running


####################################################
#  Import a binary outcome train/test set into H2O#
###################################################


# Import a sample binary outcome train/test set into H2O
train <- h2o.importFile("sepsis_train.txt")
test <- h2o.importFile("sepsis_validation.txt")


y <- "DIED"
x <- setdiff(names(train), y)
```

```
train[,y] <- as.factor(train[,y])
test[,y] <- as.factor(test[,y])

nfolds <- 10

####################################################
#  glm model #
###################################################
# Train & cross-validate a Lasso Logistic Model:
s1 <- Sys.time()
lasso2 <- h2o.glm(x = x,
                y = y,
                training_frame = train,
                family = "binomial",
                alpha = 1,
                nfolds = 10,
                stopping_metric = "MSE",
                keep_cross_validation_models=TRUE,
                seed = 1 )
t1 <- Sys.time()-s1

# Save this module for the future
saveRDS(lasso2, 'Lasso2.rds')
h2o.saveModel(object = lasso2, path = getwd(), force = TRUE)
fwrite(as.list(t1),"t1.txt")

####################################################
#  rf model #
###################################################
```

```
s2 <- Sys.time()

rf <- h2o.randomForest(x = x,
                y = y,
                training_frame = train,
                ntrees = 400,
                mtries=500,
                keep_cross_validation_predictions = TRUE,
                seed = 555)
t2 <- Sys.time()-s2
# Save this module for the future
saveRDS(rf, 'rf.rds')
h2o.saveModel(object = rf, path = getwd(), force = TRUE)
fwrite(as.list(t2),"t2.txt")

####################################################
#  XGB model   not available on windows OS#
###################################################
s3 <- Sys.time()
XGB <- h2o.xgboost(x = x,
             y = y,
             training_frame = train,
             eta = 0.15,
             max_depth = 10,
             nthread = 10,
             stopping_metric = "AUC",
             stopping_rounds = 50,
             ntrees = 400,
             nfolds=10,
```

```
                 distribution = 'bernoulli',
                 seed=1)
t3 <- Sys.time()-s3
# Save this module for the future
saveRDS(XGB, 'XGB.rds')
h2o.saveModel(object = XGB, path = getwd(), force = TRUE)
fwrite(as.list(t3),"t3.txt")

##################################################
#  ensemble model   not available on windows OS#
#################################################
s4 <- Sys.time()
ensemble <- h2o.stackedEnsemble(x = x,
                                y = y,
                                training_frame = train,
                                base_models = list(rf,XGB))
t4 <- Sys.time()-s4
# Save this module for the future
saveRDS(ensemble, 'ensemble.rds')
h2o.saveModel(object = ensemble, path = getwd(), force = TRUE)
fwrite(as.list(t4),"t4.txt")



##################################################
#  Performance#
#################################################

perf <- h2o.performance(ensemble, newdata = test)
ensemble_auc_test <- h2o.auc(perf)
```

```
#perf_gbm_test <- h2o.performance(my_gbm, newdata = test)
perf_lasso_test <- h2o.performance(lasso2, newdata = test)
perf_rf_test <- h2o.performance(rf, newdata = test)
perf_XGB_test <- h2o.performance(XGB, newdata = test)

baselearner_best_auc_test <- max(h2o.auc(perf_lasso_test),
                                 h2o.auc(perf_rf_test))

# DNN_best_auc_test <- max(h2o.auc(perf_DNN))


# print(sprintf("DNN Test AUC: %s", h2o.auc(perf_DNN)))
print(sprintf("Best Base-learner Test AUC: %s", baselearner_best_auc_test))
print(sprintf("Ensemble Test AUC: %s", ensemble_auc_test))


# [1] "Best Base-learner Test AUC:  0.76979821502548"
# [1] "Ensemble Test AUC:  0.773501212640419"
```
